# Optimized *Sambucus nigra* L., *Epilobium hirsutum* L., and *Lythrum salicaria* L. Extracts: Biological Effects Supporting Their Potential in Wound Care

**DOI:** 10.3390/antiox14050521

**Published:** 2025-04-27

**Authors:** Diana Antonia Safta, Ana-Maria Vlase, Anca Pop, Julien Cherfan, Rahela Carpa, Sonia Iurian, Cătălina Bogdan, Laurian Vlase, Mirela-Liliana Moldovan

**Affiliations:** 1 Department of Dermopharmacy and Cosmetics, Faculty of Pharmacy, “Iuliu Haţieganu” University of Medicine and Pharmacy, 12 I. Creangă St., 400010 Cluj-Napoca, Romania; diana.an.safta@elearn.umfcluj.ro (D.A.S.); catalina.bogdan@umfcluj.ro (C.B.); mmoldovan@umfcluj.ro (M.-L.M.); 2 Department of Pharmaceutical Botany, Faculty of Pharmacy, “Iuliu Haţieganu” University of Medicine and Pharmacy, 8 Victor Babes Street, 400012 Cluj-Napoca, Romania; 3 Department of Toxicology, Faculty of Pharmacy, “Iuliu Haţieganu” University of Medicine and Pharmacy, 6 L. Pasteur Street, 400349 Cluj-Napoca, Romania; anca.pop@umfcluj.ro; 4 BCBS Team (Biotechnologies et Chimie des Bioressources Pour la Santé), LIENSs Laboratory (Littoral Environment et Sociétés), UMR CNRS 7266, University of la Rochelle, 17000 La Rochelle, France; julien.cherfan@univ-lr.fr; 5 Department of Molecular Biology and Biotechnology, Faculty of Biology and Geology, Babeş-Bolyai University, 1 M. Kogalniceanu Street, 400084 Cluj-Napoca, Romania; rahela.carpa@ubbcluj.ro; 6 Department of Pharmaceutical Technology and Biopharmacy, Faculty of Pharmacy, “Iuliu Haţieganu” University of Medicine and Pharmacy, 41 Victor Babes Street, 400012 Cluj-Napoca, Romania; sonia.iurian@umfcluj.ro (S.I.); laurian.vlase@umfcluj.ro (L.V.); 7 Department 2, Faculty of Nursing and Health Sciences, “Iuliu Haţieganu” University of Medicine and Pharmacy, 4 L. Pasteur Street t, 400349 Cluj-Napoca, Romania

**Keywords:** phytocompound extraction, QbD, wound healing potential, antioxidant activity, anti-inflammatory activity, antibacterial properties

## Abstract

This study aimed to optimize the extraction of phytocompounds intended for wound care applications from three plant species, *Sambucus nigra* L. flowers and *Epilobium hirsutum* L. and *Lythrum salicaria* L. aerial parts, by using a Quality by Design approach. The effects of different extraction methods (ultra-turrax and ultrasonic-assisted extraction), ethanol concentrations (30%, 50%, 70%), and extraction times (3, 5, 10 min) were studied, and during the optimization step, the polyphenol and flavonoid contents were maximized. The phytochemical profiles of the optimized HEs (herbal extracts) were assessed using LC-MS/MS methods. The antioxidant capacity of the optimized HEs was determined using DPPH (2,2-diphenyl-1-picrylhydrazyl radical scavenging capacity) TEAC (Trolox equivalent antioxidant capacity), and FRAP (ferric reducing antioxidant power) assays, while the antibacterial activity was evaluated against *Escherichia coli*, *Pseudomonas aeruginosa*, and MSSA—methicillin-sensitive *Staphylococcus aureus* and MRSA—methicillin-resistant *Staphylococcus aureus*). Cell viability and antioxidant and wound healing potential were assessed on keratinocytes and fibroblasts. The anti-inflammatory effect was assessed on fibroblasts by measuring levels of interleukins IL-6 and IL-8 and the production of nitric oxide from RAW 264.7 cells. The major compounds of the optimized HEs were rutin and chlorogenic acid. The *Lythrum salicaria* optimized HE showed the strongest antibacterial activity, while the *Sambucus nigra* optimized HE demonstrated high cell viability. *Lythrum salicaria* and *Epilobium hirsutum* optimized HEs showed increased antioxidant capacities. All extracts displayed anti-inflammatory effects, and the *Epilobium hirsutum* optimized HE exhibited the best in vitro wound-healing effect.

## 1. Introduction

Throughout history, herbal extracts (HEs) have played an essential role in traditional medicine, particularly in dermatology, where they have been widely used for their moisturizing, anti-inflammatory, antioxidant, antimicrobial, and regenerative properties [1,2,3,4]. The therapeutic benefits of bioactive phytocompounds in skin health are deeply rooted in ancient medical practices, where HE-based formulations were commonly used to treat wounds, burns, infections, and various skin conditions. As one of their most significant applications in ethnopharmacology is their role in wound care, modern scientific advances continue to validate and expand this traditional use [5,6,7].

Wound healing is a complex, multi-phase process involving four overlapping and dynamic stages: hemostasis, inflammatory phase, proliferative phase, and maturation or remodeling phase [3,8,9,10]. The active ingredients used in wound care should address the needs of each phase, such as reducing the bleeding and inflammation and promoting tissue repair by various mechanisms (e.g., angiogenesis, stimulation of growth factors, increasing the reepithelization, cell migration, and wound closure) [3,11]. Moreover, the antioxidant property has been demonstrated to be very important, as oxidative stress can damage skin cells and tissues, prolonging the healing process [3,12,13,14,15,16]. In addition, antimicrobial effects are crucial, since infections significantly delay the healing and lead to complications like chronic wounds or sepsis [17,18,19]. Subsequently, the growing issue of antibiotic resistance demands novel antimicrobial ingredients that can effectively combat infections without contributing to resistance [20,21,22].

Up to now, HEs seem to meet all the criteria concerning the antioxidant, antimicrobial, anti-inflammatory, and wound-healing effects, together with their safety, cost efficacy, and easy availability. The phytocompounds from HEs may work synergistically and complementarily to address the different stages of wound healing and significantly improve the healing outcome [3,7]. Moreover, due to the presence of multiple and complex phytocompounds in HEs, the development of antimicrobial resistance is therefore reduced [20]. Despite these advantages, their inherent variability is a drawback in their use. Thus, an efficient and systematic extraction of phytocompounds from plant materials is important to maximize their bioactive yield, ensuring reproducibility and efficacy. Consequently, to optimize the extraction of the active phytocompounds, the Quality by Design (QbD) approach may be used. The QbD approach includes all the elements that will lead to a quality product and a production process capable of constantly providing the required efficacy and safety [23,24,25,26].

The present study aimed to optimize, with the QbD approach, the extraction of the phytocompounds from three plant materials commonly found in European spontaneous flora (*Sambucus nigra* L. flowers, *Epilobium hirsutum* L. aerial parts, and *Lythrum salicaria* L. aerial parts) and to investigate the biological effects of the optimized herbal extracts (OHEs), namely, the antioxidant, antimicrobial, anti-inflammatory, and wound-healing effects.

*Sambucus nigra* L. (Adoxaceae family), commonly known as elderberry, has been extensively used in traditional European medicine, with its leaves, fruits, and flowers applied topically for the treatment of wounds and various skin and mucosal disorders [27,28]. These effects have been attributed to its anti-inflammatory, antioxidant, antiviral, antibacterial, and analgesic effects [29,30,31,32,33]. However, despite this traditional use, scientific studies investigating the wound-healing potential of *Sambucus nigra* HEs—particularly from the inflorescences—remain scarce [34,35]. Species of the genus *Epilobium* (Onagraceae family), commonly known as willowherbs, have been widely used in folk medicine, mainly for alleviating the symptoms of benign prostatic hyperplasia. Additionally, they have also been employed for the treatment of wounds, burns, and inflammatory skin conditions. This ethnomedicinal application has gained scientific support in recent years [36,37,38,39], with studies highlighting the anti-hyaluronidase, anti-collagenase, and antioxidant activities of these species [36,40]. Although *Epilobium hirsutum* L. has demonstrated relevant anti-inflammatory and antioxidant effects [24,41], its potential role in the complex process of wound healing remains poorly documented, particularly in comparison with the more widely studied *E. angustifolium* [37,38,42]. *Lythrum salicaria* L. (Lythraceae family), commonly known as purple loosestrife, has been used in traditional medicine for treating diseases with an inflammatory background, showing also astringent, analgesic, antibacterial, and wound-healing effects [43,44]. Previous studies have demonstrated their antioxidant, anti-inflammatory, and burn wound-healing activities with promising results [45,46]. Moreover, *Lythrum salicaria* HE may contribute to the maintenance of skin homeostasis through pro-differentiating and skin barrier-strengthening effects [47], which are particularly relevant in the context of tissue regeneration and repair.

These three HEs were selected for the present study based on their historical use in the topical treatment of skin lesions combined with the relative scarcity of modern scientific investigations validating their wound-healing properties. While each species has shown individual pharmacological potential, their comparative evaluation—both phytochemical and biological—remains limited. Given the presence of bioactive compounds with known relevance to the different phases of wound repair (e.g., antioxidants, anti-inflammatories, antimicrobials, wound-healing agents), it is reasonable to hypothesize that these extracts may act synergistically or complementarily to promote skin regeneration. Therefore, the current study aims to provide a comprehensive analysis of the chemical composition and biological activity of the *Sambucus nigra* (inflorescences), *Epilobium hirsutum*, and *Lythrum salicaria* (aerial parts) HEs. By scientifically validating their traditional use and exploring their potential as wound-healing agents, this work seeks to contribute to the development of new natural formulations for skin health. In the present study, the effects of different extraction methods (ultra-turrax-assisted extraction—UTE, ultrasonic-assisted extraction—USE), different ethanol concentrations (30%, 50%, 70%), and different extraction times (3, 5, 10 min) on these plant materials were studied. To better understand how the variation factors in the OHEs’ preparation can affect their attributes, the Design of Experiment (DoE) was employed. Finally, the total polyphenol and flavonoid contents of each extract were maximized to achieve the optimal extraction. Thus, the phytochemical profiles and the antioxidant and antimicrobial activities of the OHEs were investigated. The in vitro biological effects of the OHEs on cell cultures were studied by investigating cell viability, antioxidant capacity, anti-inflammatory activity, and wound-healing assay. To the best of our knowledge, this is the first study that reported the ultra-turrax-assisted extraction of phytocompounds from *Sambucus nigra* and *Lythrum salicaria*. By optimizing the extraction process, this research aims to maximize the therapeutic potential of polyphenols, contributing to more effective wound-healing solutions. While previous studies focused on assessing various biological activities, this study integrates a DoE approach to optimize extraction parameters, correlating them with detailed in vitro analyses of biological activities for a comprehensive characterization of the extracts. To the best of our knowledge, this the first report on the effects of *Sambucus nigra* flower, *Lythrum salicaria* and *Epilobium hirsutum* HEs on the HaCaT cell line and the first research investigating the effects of *Lythrum salicaria* and *Epilobium hirsutum* HEs on RAW 264.7 cells. Moreover, this seems to be the first study to evaluate the wound-healing potential of *Lythrum salicaria* aerial parts, *Epilobium hirsutum* aerial parts HEs, and *Sambucus nigra* flower HE through scratch assay.

## 2. Materials and Methods

### 2.1. Chemicals and Reagents

The following reagents were purchased from Sigma Aldrich (Sigma Aldrich Chemie GmbH, Schnelldorf, Germany): ethanol, methanol, ferric chloride, 6-hydroxy-2,5,7,8-tetramethylchromane-2-carboxylic acid (Trolox), 2,2-Diphenyl-1-Picrylhydrazyl (DPPH), 2,4,6-Tripyridyl-S-triazine (TPTZ), 2,2′-azino-bis (3-ethylbenzothiazoline-6-sulfonate) (ABTS), dimethyl sulfoxide (DMSO) (≥99%), hydrogen peroxide (H_2_O_2_) 30% solution, fetal bovine serum (FBS), resazurin, MTT (3-(4,5-Dimethylthiazol-2-yl)-2,5-Diphenyltetrazolium Bromide), 2,7 dichloro-fluorescein diacetate (DCFH-DA), N-acetyl-l-cysteine (≥99%), sulfanilamide, N-(1-napthyl)ethylenediamine dihydrochloride (NED), phosphoric acid, dexamethasone, and lipopolysaccharides isolated from *E. coli*. Other reagents used were: Folin–Ciocâlteu reagent (Chempur, Piekary Śląskie, Poland), acetic acid and sodium carbonate (International Laboratory, Cluj-Napoca, Romania), aluminum chloride (Thermo Fisher Scientific Inc., Waltham, MA, USA). All solvents were of LC grade, and each of the reagents used was of analytical grade. For the spectrophotometric assays and (LC-MS/MS) analysis, β-sitosterol was purchased from Carl Roth (Karlsruhe, Germany), ferulic acid and gallic acid from Merck (Darmstadt, Germany), (+)-δ-tocopherol from Supelco (Bellefonte, PA, USA), and all other standards were acquired from Sigma Aldrich (Sigma Aldrich Chemie GmbH, Schnelldorf, Germany): apigenin, brassicasterol, caffeic acid, 4-O-caffeoylquinic acid, (+)-catechin, caftaric acid, campesterol, chlorogenic acid, *p*-coumaric acid, ergosterol, (-)-epicatechin, gentisic acid, hyperoside (quercetin, 3-d-galactoside), isoquercitrin (quercetin 3-β-d-glucoside), kaempferol, kaempferol-3-rhamnoside, luteolin, myricetin, protocatechuic acid, quercetin, quercitrin (quercetin 3-rhamnoside), rutoside (quercetin-3-O-rutinoside), stigmasterol, syringic acid, (+)-α-tocopherol, (+)-γ-tocopherol, and vanillic acid. For microbiological assay, the bacterial strains *Escherichia coli* ATCC 25922, *Pseudomonas aeruginosa* ATCC 27853, *Staphylococcus aureus* methicillin-sensitive (MSSA) ATCC 25923, and *Staphylococcus aureus* methicillin-resistant (MRSA) ATCC 700699 were acquired from Microbiologics Inc. (St. Cloud, MN, USA). Gentamicin (CN10) was purchased from Oxoid, Thermo Scientific Inc. (Waltham, MA, USA). The immortalized human keratinocytes (HaCaT) were purchased from CLS Cell Lines Service (Eppelheim, Germany), while the human foreskin fibroblasts (BJ) and the mouse macrophage cell line RAW 264.7 were purchased from American Type Culture Collection (Manassas, VA, USA). Dulbecco’s Modified Eagle Medium (DMEM) and phosphate-buffered saline (PBS) were purchased from Gibco (Life Technologies Limited, Paisley, UK). Human IL-8 and IL-6 ELISA Kits were purchased from Invitrogen (Thermo Fisher Scientific Inc. Waltham, MA, USA).

### 2.2. Harvesting of Plant Material and Preparation for Extraction

The three plant materials were harvested during flowering stage from Romanian flora, as presented in Table 1. The plant species were authenticated by botany Assistant Professor Ana Maria Vlase and Professor Mircea Tămaș from Department of Pharmaceutical Botany, Faculty of Pharmacy, Iuliu Hațieganu University of Medicine and Pharmacy, Cluj-Napoca. The voucher specimens were deposited for each species in the Herbarium of this department and numbered as presented in Table 1.

The vegetal material of each species was separated and air-dried at room temperature safe from sunlight. The plant material was ground in a coffee grinder (Selecline CG9140-GS, 60 g, 150 W, Croix, Hauts-de-France, France for approximately 3 min, and the powder was sifted through a 200 µm Retsch sieve (Retsch GmbH, Haan, Germany) [24].

### 2.3. QbD Approach for Extract Development and Optimization

#### 2.3.1. Quality Target Product Profile (QTPP) Definition

The QTPP was established by setting the critical quality attributes (CQAs), critical material attributes (CMAs), and critical process parameters (CPPs) [23,26,48]. Furthermore, acceptance criteria were set to guide the development process.

#### 2.3.2. Risk Analysis

A risk analysis was used to screen the CMAs and CPPs with a possible impact on the CQAs and to identify appropriate process control points [48].

The guideline ICH Q9 “Quality Risk Management” provides a list of risk management tools, out of which Ishikawa’s fishbone diagram and failure mode and effects analysis (FMEA) are among the most common. An Ishikawa diagram filled in by an interdisciplinary team aimed to identify and examine the risk factors (CMAs or CPPs) related to the preparation of the OHEs. Further, FMEA approach, based on the evaluation of three criteria—frequency of occurrence (O), severity of consequences (S), and difficulty of detection (D)—was used for factor ranking. Giving a score from 1 to 5, each of these criteria was assigned to each variation factor from the Ishikawa diagram as explained below. The occurrence (O): 5—frequent, 4—probable, 3—occasional, 2—remote, 1—improbable; the severity (S): 5—catastrophic, 4—critical, 3—serious, 2—minor, 1—negligible; detectability (D): 5—hard to detect, 4—low chance of detection, 3—moderately detectable, 2—highly detectable, 1—easily detectable. The risk priority number (RPN) was obtained by multiplying these three attributes [23].

#### 2.3.3. Design of Experiment (DoE)

To obtain a deeper understanding of the variables with the highest risk priority number (RPN), DoE was employed as a risk control and reduction tool. The extraction process for each plant material was evaluated using a screening D-optimal experimental design (Modde 13.1 software, Sartorius Stedim, Göettingen, Germany) to identify the effects of the highest-ranked failure mode and effects analysis (FMEA) variables on extraction efficiency. The input variables included relevant extraction parameters (extraction method—X1, extraction time—X2, ethanol ratio in the extraction solvent). The output variables determined were total polyphenol content (Y1) and total flavonoid content (Y2), as summarized in Table 2. The same general design matrix was applied to each plant material, resulting in 3 DoE models, each consisting of 10 individual runs and 3 center points.

#### 2.3.4. Extraction Methods and Bioactive Compounds Screening

According to the DoE, different extraction methods, times, and solvents were used, as described above in Table 2. For all the HEs, a plant material/solvent ratio of 1:10 (*m*/*v*) was used. The solvent was a mixture of ethanol (30%, 50%, 70%) and distilled water. UTE was performed by using the ultra-turrax homogenizer with the W 45MA fixture at 4000 rpm (model T 18, IKA Labortechnik, Staufen, Germany). USE was carried out with an ultrasonic bath (Sonorex Super RK 100 H, Bandelin Electronic GmbH & Co. KG, Berlin, Germany). The extraction was performed at room temperature for 3, 5, or 10 min. After extraction, the samples were centrifuged for 8 min at 10,000 rpm at 22 ± 0.5 °C using a refrigerated centrifuge (Sigma Laborzentrifugen GmbH, Osterode am Harz, Germany). The supernatant was recovered and stored in a refrigerator at 5 ± 2 °C for further analysis.

For the evaluation of the biological activities on cell cultures, the OHEs were concentrated using a rotary evaporator coupled with vacuum pump (HEI-VAP Advantage Rotary evaporator HL/G1 coupled with Rotavac valve control, Heidolph, Schwabach, Germany) until complete ethanol evaporation and then subjected to lyophilization using SP Scientific Virtis AdVantage 2.0 BenchTop Freeze Dryer, Advantage Plus EL-85 (American Laboratory Trading Inc., East Lyme, CT, USA). During the lyophilization, the following parameters were used: freezing for 24 h at −55 °C and afterward for 48 h at −25 °C under pressure of 200 mTorr. The lyophilized OHEs were stored in a refrigerator at 5 ± 2 °C for further analysis [24,25,49].

The total polyphenol content (TPC) was quantified using the Folin–Ciocâlteu assay following a previously validated method [24,50]. HE samples were diluted 1:10 with distilled water, and then mixed with Folin–Ciocâlteu reagent and 6% Na_2_CO_3_ solution in Eppendorf tubes. The reaction mixture was incubated in the dark at room temperature for 30 min to allow for color development. The calibration curve was constructed using gallic acid as a reference standard, with a concentration range of 10–100 µg/mL (R^2^ = 0.9917, y = 0.0053x + 0.0589). The results were expressed as µg gallic acid equivalents (GAE) per mL of each HE.

The total flavonoid content (TFC) was determined using a quercetin-based colorimetric assay following a previously validated methodology [24,51]. The HE samples were appropriately diluted—1:10 for *Sambucus nigra* and 1:5 for *Lythrum salicaria* and *Epilobium hirsutum.* The quantification was performed using quercetin as the reference standard, with a calibration curve over a concentration range of 20–150 µM (R^2^ = 0.9956, y = 1.8184x + 36.6140). The results were expressed as µM quercetin equivalents (QAEs) per each HE.

The Flow Injection Analysis (FIA) method was employed for the rapid and efficient quantification of TPC and TFC, utilizing an HPLC system equipped with a diode array detector (DAD). Unlike conventional chromatographic techniques, FIA operates without a separation column, allowing direct injection of the sample into the system for immediate detection and quantification. The analysis was performed using an Agilent Technologies 1100 HPLC Series system (Agilent, Santa Clara, CA, USA), with an injection volume of 5 µL per sample. The mobile phase consisted of distilled water. Detection wavelengths were set at 760 nm for TPC and 370 nm for TFC. All experiments were performed in triplicate (n = 3), and results are presented as mean value ± standard deviation (SD).

### 2.4. Characterization of OHEs

After the optimization step, the phytochemical profiles of the three OHEs were assessed by liquid chromatography–tandem mass spectrometry (LC-MS/MS) with five distinct analytical methods, which were previously validated [24,25]. The following equipment was used: Agilent Technologies 1100 HPLC Series system (Agilent, Santa Clara, CA, USA) equipped with auto sampler, column thermostat, binary gradient pump, degasser, and UV detector. The system was coupled with a mass spectrometer from Agilent, model Ion Trap 1100 SL (LC/MSD Ion Trap VL, Agilent, Santa Clara, CA, USA).

DataAnalysis (v5.3) and ChemStation (vB01.03) software from Agilent (Santa Clara, CA, USA) were used for chromatographic data acquisition and interpretation. Further methodological details are available in previous publications [24,25,52].

#### 2.4.1. Identification and Quantification of Polyphenolic Compounds

Polyphenolic compounds in the OHEs were identified and quantified using two validated LC-MS analytical methods. The first method targeted 23 polyphenols using a Zorbax SB-C18 reverse-phase column (100 mm × 3.0 mm i.d., 3.5 μm, Agilent Technologies, Santa Clara, CA, USA) for separation with a binary elution gradient. The mobile phase consisted of methanol and 0.1% acetic acid. The flow rate was 1 mL/min, column temperature 48 °C, and injection volume 5 µL. Detection was performed in UV mode at 330 nm for polyphenolic acids and 370 nm for flavonoids, while electrospray ionization–mass spectrometry (ESI-MS) in negative mode was further used for confirming the identity of targeted bioactive compounds.

The second LC-MS analytical method aimed to identify and quantify eight additional polyphenols (epicatechin, catechin, syringic acid, gallic acid, protocatechuic acid, vanillic acid, epicatechin gallate (ECG), and epigallocatechin gallate (EGCG)). Chromatographic separation followed a similar approach, and it was employed using the above-mentioned equipment and chromatographic column. The same LC-MS conditions were applied using UV and MS spectra comparisons for compound identification and calibration curves of individual compounds for quantification [24,25,52].

The equations of the calibration curves and the limits of detection (LOD) and quantification (LOQ) are presented in Table S1 (for polyphenols analyzed with the first analytical method) and in Table S2 (for polyphenols analyzed with the second analytical method).

#### 2.4.2. Identification and Quantification of Phytosterols

Phytosterols were analyzed using a validated LC-UV-MS/MS method. Chromatographic separation was achieved through isocratic elution, with a mobile phase consisting of acetonitrile and methanol (90:10, *v*/*v*). Detection was performed in positive ionization mode using an Atmospheric Pressure Chemical Ionization (APCI) source. Identification was based on spectral data and retention time matching with external standards for ergosterol, brassicasterol, stigmasterol, campesterol, and β-sitosterol. Further details on this method can be found in previous publications [24,52]. The results were expressed as micrograms of phytosterol per milliliter of each OHE (mean values ± SD, n = 3). The equations of the calibration curves, LOD, and LOQ are presented in Table S3.

#### 2.4.3. Identification and Quantification of Tocopherols

Tocopherol (α, γ, δ) content in the optimized extracts was evaluated through LC-MS/MS following a validated protocol. The analysis was performed using an isocratic elution system with water/methanol (7:93, *v*/*v*) as the mobile phase. Detection was carried out in negative ionization mode using APCI-MRM (Multiple Reaction Monitoring). Tocopherol standards were prepared and analyzed under the same conditions for quantification. Further methodological details are available in previously published studies [24,52]. The results were expressed as nanograms tocopherol per milliliter of each OHE (mean values ± SD, n = 3). The equations of the calibration curves, LOD, and LOQ are presented in Table S4.

#### 2.4.4. Identification and Quantification of Procyanidins

The procyanidin content was assessed using LC-MS/MS applying a gradient elution system with methanol and 0.1% acetic acid in water. Mass spectrometric detection was conducted in negative ionization mode, with fragmentation patterns confirming the presence of procyanidin A1, B1, and B2. Calibration curves were performed for quantification, and further details regarding the chromatographic conditions and MS parameters are available in prior studies [25]. The equations of the calibration curves, LOD, and LOQ are presented in Table S5.

### 2.5. Evaluation of Biological Activities of the OHEs

#### 2.5.1. Antioxidant Activity of the OHEs

##### 2,2-Diphenyl-1-Picrylhydrazyl (DPPH) Radical Scavenging Capacity

The antioxidant potential of the OHEs was evaluated using the DPPH assay following an established methodology. OHEs were diluted 1:200 with distilled water and mixed with DPPH reagent, incubated for 30 min in the dark, and afterward, the absorbance was measured at 517 nm using a UV-Vis spectrophotometer (Specord 200 Plus, Analytik Jena, Jena, Germany). The scavenging capacity was calculated in relation to a Trolox calibration curve (R^2^ = 0.9950, y = 0.0038x + 0.0377), with results expressed as mg of Trolox equivalents (TEs) per mL of each OHE (mean values ± SD, n = 3) [24,52,53].

##### Ferric Reducing Antioxidant Power (FRAP) Assay

The FRAP assay was conducted using a reagent mixture containing acetate buffer, 2,4,6-Tri(2-pyridyl)-s-triazine (TPTZ), and FeCl_3_. OHEs were diluted 1:100 with distilled water, incubated with the reagent mixture for 30 min in the dark, and the absorbance was measured at 593 nm using UV-VIS spectrophotometer (Specord 200 Plus, Analytik Jena, Jena, Germany). The calibration curve was prepared with Trolox (R^2^ = 0.9834, y = 0.4216x + 0.1534), and results were reported as Trolox equivalents (TEs) in mM of each OHE (mean values ± SD, n = 3) [52].

##### Trolox Equivalent Antioxidant Capacity (TEAC) Assay

The TEAC assay was assessed by mixing the diluted OHEs (1:200 with distilled water) with acetate buffer and 2,2′-azino-bis (3-ethylbenzothiazoline-6-sulfonate) (ABTS) reagent followed by 5 min incubation at room temperature. Absorbance was recorded at 660 nm, and antioxidant capacity was determined based on a Trolox standard curve (R^2^ = 0.9972, y = 0.264x + 0.0048), with results expressed as Trolox equivalents (TEs) in mM of each OHE (mean values ± SD, n = 3) [24,52].

#### 2.5.2. Antibacterial Activity of the OHEs

The following microorganisms were used in this experiment: *Escherichia coli* ATCC 25922, *Staphylococcus aureus* MSSA ATCC 25923, *Staphylococcus aureus* MRSA ATCC 700699, and *Pseudomonas aeruginosa* ATCC 27853. The antibacterial activity was tested qualitatively through a qualitative disk diffusimetric method, which is considered a reliable method with large applicability in microbiological practice to test the efficacy of most antimicrobial agents [54,55]. The diameter of the inhibition area was measured after the incubation of bacterial strains with OHEs for 24 h at 37 °C. The solvent, 70% hydroethanolic solution (EtOH 70%), served as negative control, while gentamycin served as positive control. The diameter of the inhibition area was correlated with the sensitivity of bacteria to the tested sample [54,56].

The minimum inhibitory concentration (MIC) method was used for the quantitative assessment of the same bacterial strains. The evaluation was performed according to the adapted EUCAST (European Committee on Antimicrobial Susceptibility Testing) protocols [57]. Liquid Mueller–Hinton (MH) broth medium was used to dilute the extracts in a 2-fold serial system, in 10 consecutive wells, in 96-well titer plates. The total broth volume was adjusted to 200 µL/well, with 180 µL from the HE and 20 µL from the microbial suspension. Positive (MH broth and bacterial inoculum) and negative (MH broth and HE) controls were also used. Three repetitions were run for each HE. The plates were incubated at 37 ± 2 °C for 24 h. The MIC values were represented by the lowest concentration of the extract able to inhibit the growth of the bacterial strains compared with the positive control.

#### 2.5.3. In Vitro Cell Culture Assays for the OHEs

##### Preparation of OHE Solutions

A 100 mg/mL stock solution in dimethyl sulfoxide (DMSO) was obtained by dissolving each previously lyophilized OHE. The testing solutions were prepared by diluting the stock solution with DMSO, and they were further diluted in the cell culture medium to achieve the necessary concentrations, which ranged from 1 to 400 µg/mL.

##### Cell Cultures

HaCaT, BJ, and RAW 264.7 cells were maintained in DMEM supplemented with 10% fetal bovine serum (FBS) and 10% antibiotics (penicillin + streptomycin), with the difference that the HaCaT cell line had extra 1% sodium pyruvate. Cells were cultured in a humidified incubator at 37 °C with 5% CO_2_, and the medium was changed every 2 days. Further, cells were seeded in 96-well sterile plates and allowed to adhere to the substrate for 24 h.

##### Cell Viability

Two cell viability tests were performed. The Alamar Blue assay was used for HaCaT and BJ cell lines, while the MTT assay (3-(4, 5-dimethylthiazolyl-2)-2, 5-diphenyltetrazolium bromide) was chosen for RAW 264.7 cell line. The evaluation of cell viability on HaCaT and BJ cell lines was performed after a 24 h incubation period, with the three OHEs at doses ranging from 1 to 400 μg/mL, as previously described [58]. Following the exposure, cells were washed with PBS and further incubated for 2–3 h with a 50 mM solution of resazurin prepared in medium. Using this assay, the ability of cells to metabolically transform resazurin, a non-fluorescent substance, into resorufin, a fluorescent product, was evaluated. The fluorescence, which is proportional with the number of metabolically active cells, was measured at λ_excitation_ = 530/25, λ_emission_ = 590/35, using Synergy 2 Multi-Mode Microplate Reader (BioTek Instruments, Winooski, VT, USA). Cells exposed to medium with DMSO were used as negative control. Results were presented as relative values in comparison with the negative control (100%). RAW 264.7 cells were seeded in 96-well plates and incubated overnight at 37 °C with 5% CO_2_. Cells were exposed to different concentrations of OHEs, ranging from 1 to 400 μg/mL, for 24 h, after which the medium was replaced with a fresh medium containing 10% MTT and incubated for 4 h. The MTT-containing medium was discarded, and DMSO (100 μL/well) was added to dissolve the formazan crystals. Absorbance at 570 nm was detected by Synergy 2 Multi-Mode Microplate Reader [59]. For the next experiments, only the non-toxic concentrations were tested.

##### Antioxidant Activity in Cell Cultures

The ability of the three OHEs to protect against oxidative stress was evaluated on the cell cultures using the 2,7 dichloro-fluorescein diacetate (DCFH-DA) assay, as previously described [60]. Following cellular integration, intracellular esterases hydrolyze the non-fluorescent colored DCFH-DA to dichlorodihydrofluorescein (DCFH), which remains inside the cellular compartment. DCFH is oxidized by ROS to a fluorescent molecule that is proportional to the amount of intracellular ROS. The fluorescence was measured using Synergy 2 Multi-Mode Microplate Reader at an λ_excitation_ = 485/20 and λ_emission_ = 528/20. The study was performed in the presence or absence of a pro-oxidant (H_2_O_2_) (Hanks’ Balanced Salt Solution (HBSS) for non-stimulated conditions, and 250 µM H_2_O_2_ in HBSS for stimulated conditions). The antioxidant activity of OHEs was compared with N-acetylcysteine (NAC) treatment as positive control (20 mM solution). Results were presented as relative values in comparison with the negative control (100%).

##### Anti-Inflammatory Activity in Cell Cultures

The anti-inflammatory activity of the OHEs was evaluated using ELISA kits by measuring the levels of two pro-inflammatory cytokines, namely IL-6 and IL-8, in the cell culture supernatant. The cells were exposed to 100 ng/mL bacterial lipopolysaccharide (LPS) and three biocompatible concentrations of the OHEs. After the 24 h exposure, the supernatant was collected. The concentrations of IL-6 and IL-8 were measured using commercially available ELISA kits according to the manufacturer’s instructions (Invitrogen, Thermo Scientific, USA) using Synergy 2 Multi-Mode Microplate Reader.

##### Measurement of Nitric Oxide (NO) Production

RAW 264.7 cells were pretreated with non-toxic concentrations of each OHE for 6 h, and then stimulated for 18 h with or without 1 µg/mL LPS at 37 °C in an incubator with 5% CO_2_. The NO level in the culture supernatants was measured using Griess reagents by adding 50 µL 1% sulfanilamide and 50 µL 0.1% N-(1-napthyl) ethylenediamine dihydrochloride in 5% phosphoric acid to 100 µL of culture supernatant in each well and incubating them at room temperature for 15 min in the dark. Subsequently, absorbance at 540 nm was measured with Synergy 2 Multi-Mode Microplate Reader. A standard curve was prepared using NaNO_2_ as a standard solution in the same manner, and it was used to calculate the concentration of NO [61]. Dexamethasone at a concentration of 1 µM was used as positive control. All the experiments, unless specified otherwise, were done using three biological replicates, each one including three technical replicates.

##### Wound Healing Assay in Cell Cultures

The scratch assay was assessed to investigate the in vitro wound healing determined by the OHEs. BJ and HaCaT cells were seeded in 96-well plates in DMEM medium with 10% FBS to reach the confluency, and then, wounds were performed using 10 µL sterile pipette tips. After that, the cells were washed with PBS and further treated with OHE in highest biocompatible concentration (H) for each OHE. At least 3 images of each well were taken using an inverted Zeiss Axio Observer Z1 microscope (Zeiss, Jena, Germany) with AxioCam Icc Rev.4 CCD camera (1.4 megapixels, Zeiss) and processed by the ZEN 2.3 lite software at T_0_ and after 24 h to observe the changes that occurred in cell migration. The images were then processed using ImageJ 1.54d software (National Institutes of Health, Bethesda, MD, USA), and the cell migration rate and wound closure percent were calculated according to the following equations:

$$Wound\;closure\left(\%\right)=\frac{\left(A_{t0}-At_{\Delta t}\right)}{A_{t0}}\times 100$$

$$Cell\;migration\;rate(\frac{mm}{h})=\frac{\left(W_{i}-W_{f}\right)}{t}$$

where W_i_ is the average of the initial wound width, W_f_ is the average of the final wound width (mm), and t is the time of the assay (hours). Additionally, A_t0_ is the initial wound area and A_tΔt_ is the wound area after Δt hours of the initial scratch, both in mm^2^ [62].

### 2.6. Statistical Analysis

All the results were presented as mean ± standard deviation (SD) and were analyzed in triplicate. Modde 13.1 software was used to perform data processing of the results from the DoE together with their graphical representations, and the data were statistically analyzed by ANOVA test. For in vitro cell culture assays, experimental data are presented as mean values ± standard deviations (SD) of three biological replicates. The antibacterial activity and in vitro cell culture assays were statistically analyzed using a One-Way Analysis of Variance (ANOVA) using the GraphPad Prism 10.3.1 software (La Jolla, CA, USA), and results were considered statistically different if *p* values were lower than 0.05. For in vitro cell culture assays, graphical representation was also performed by using GraphPad Prism 10.3.1 software.

## 3. Results and Discussion

### 3.1. QbD Approach for Extract Development and Optimization

#### 3.1.1. Definition of QTPP

To develop the OHEs with the desired quality using the QbD approach, the QTPP was established. It is based on the following quality indicators: high efficiency, low toxicity, and the possibility of long-term use in therapy [48]. As previously reported, the bioactive phytocompounds represented by polyphenols and flavonoids may have a significant impact on the stimulation of effective wound healing and may prevent impaired healing [3,31,63,64]. Therefore, the aim of the process was to obtain high-quality OHEs rich in polyphenols and flavonoids and thus with pronounced antioxidant, antimicrobial, anti-inflammatory, and wound-healing properties (Table 3).

#### 3.1.2. Results of the Risk Analysis

Aiming for the quality attributes revealed by the QTPP, the materials and the available extraction processes were screened for critical characteristics and parameters that could impact the quality. For a thorough risk source identification, they were divided into four categories, materials, process, equipment, and methods-related risks, and for each of them, the possible variables were identified, as shown in the Ishikawa diagram (Figure S1). FMEA is a tool that allows risk ranking; all the variables previously identified were included and analyzed, and results are presented in Table S6. The scores were attributed for each variation factor according to its occurrence (O), severity (S), and detectability (D), and the greatest scores were registered for the extraction solvent, method of extraction, and time of extraction [23,26]. The extraction process’s effectiveness is known to be highly dependent on the extraction solvent. An ideal solvent should have low toxicity and should also exhibit a preservative effect on the extract. It is well known that ethanol is a safe alternative to other organic solvents and serves effectively as a polar solvent for polyphenol extraction. Similarly, water, the solvent most used for plant extraction, offers advantages in the recovery of phytocompounds from the chosen plant materials. Both ethanol and water contain hydroxyl groups, allowing them to form hydrogen bonds with the phytocompounds, making their ratios in a hydroethanolic mixture a critical factor that influences the quality of the final extract [65]. Thus, we chose mixtures of ethanol and water in different ratios as the extraction solvents. Extraction yield and biological activities not only are critically dependent on the solvent, but also on the extraction methods, with all their parameters or characteristics. In this study, two non-conventional extraction methods, USE and UTE, were selected for comparison. To ensure a consistent comparison, the rest of the parameters were constant within the DoE. To the best of our knowledge, these two extraction methods have not been compared previously. USE employs an elastic mechanical wave to increase cell wall permeability and to induce cavitation, significantly reducing extraction time and temperature, lowering solvent usage, and improving yield compared with conventional extraction methods [65]. The ultra-turrax is a high-speed shearing homogenizer widely used for homogenizing immiscible liquid/liquid systems and for dispersing raw powder crystals into a liquid phase. Due to the very strong mass transfer forces and speed that the ultra-turrax can impart on the material, it was hypothesized that high-speed stirring could be appropriate for the extraction of the phytocompounds from the plant materials dispersed in a suitable solvent [66], and good results were previously reported [24,67]. Additionally, extraction time plays an important role in determining the quality of the extracts. While longer times can increase extraction yield, extended exposure to heat can lead to oxidation and degradation of the unstable or thermolabile phytocompounds [65]. Therefore, to obtain the greatest outcomes, different extraction times were also tested.

In summary, the key parameters considered for the DoE were the extraction method, the ethanol ratio in the solvent, and the extraction time. Our goal was to minimize the risk of failure and assess the impact of modifying these parameters. To guarantee that the HEs were consistently of high quality and composition, each of these was investigated.

#### 3.1.3. Summary of Fit

For each response, Multiple Linear Regression was used to generate mathematical models that best fit the experimental data. As observed in Table 4, overall, the results of the data fitting were acceptable, except for the results for Y1 (TPC) for *Sambucus nigra* HE, which is not further discussed; the optimization was made by maximizing only Y2 (TFC). The results showed the minimum R^2^ values of 0.589, the minimum Q^2^ values of 0.335, and the maximum difference between these two parameters of 0.30. The model validity values ranged between 0.443 and 0.795, and reproducibility between 0.835 and 0.999. R^2^ represents the percentage of the variation in the response explained by the model (the fit between the data and the model), while Q^2^ is defined as the percentage of the variation in the response predicted by the model (the predictive power of the model). A good model is shown by high values for these two parameters and by reduced differences between these two values. The model validity parameter indicates whether the appropriate model type has been developed, and the reproducibility shows the response variation under identical conditions. High values of the model validity and reproducibility show a high significance of the chosen model [23]. Table 3 shows that the *p*-values were below 0.05, while the lack of fit values were above 0.05, except for the results for Y1 (TPC) for *Sambucus nigra* HE. These values indicated statistically significant models.

#### 3.1.4. The Influences of Extraction Conditions on the TFC and TPC of the HEs

In Table 5, the matrix of the DoE is presented with the results of the TFC and TPC of the HEs.

**Figure 1:**
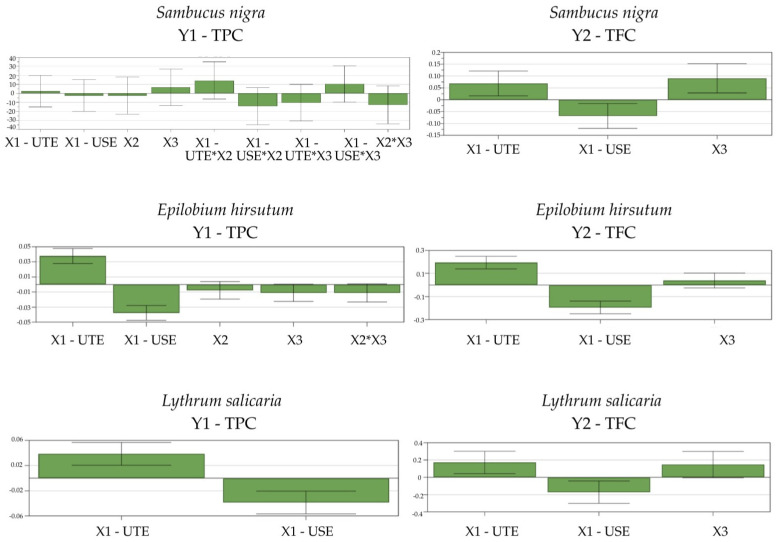
The influence of extraction conditions on TPC (total phenolic content) and TFC (total flavonoid content) of the vegetal extracts of *Sambucus nigra*, *Lythrum salicaria*, and *Epilobium hirsutum*, presented as centered and scaled coefficient plots. Y1-TPC, expressed as μg gallic acid equivalent (GAE)/mL HE; Y2-TFC, expressed as μM quercetin equivalent (QAE)/HE; UTE—ultra-turrax-assisted extraction; USE—ultrasound-assisted extraction, X1—extraction method, X2—extraction time, X3—ethanol ratio in the extraction solvent.

For *Sambucus nigra* HE, the TPC ranged between 4749.95 and 5282.38 μg GAE/mL HE, while the TFC varied between 1032.13 and 3342.00 as μM QAE/HE. For *Lythrum salicaria* HE, the TPC ranged between 5178.39 and 7246.25 μg GAE/mL HE, and the TFC varied from 148.78 to 1023.92 μM QAE/HE. For *Epilobium hirsutum* HE, the TPC varied from 5238.74 to 6705.09 μg GAE/mL HE, while the values of TFC ranged between 522.73 and 2068.84 μM QAE/HE, as shown in Table 4. The model coefficients, shown in Figure 1 as histograms, indicate the effects the extraction conditions had on the extraction yields of the phytocompounds. The recovery of TPC and TFC for all three HEs was increased by using UTE compared with USE, which decreased their extraction. To the best of our knowledge, this is the first paper exploring the UTE of phytocompounds from *Sambucus nigra* HE and the UTE and USE from *Lythrum salicaria* HE, respectively. The positive influence of UTE upon the extraction yield of TPC and TFC from *Epilobium* species was previously observed by Vlase et al. [24]. The better extraction yield using UTE compared with USE can be explained by the mechanical force employed in homogenization, which can reduce the particle size of the plant material, improving solvent penetration into the plant cells and aiding the extraction of intracellular substances. The processing time did not affect the extraction yield for any of the plant materials, which could be due to the narrow range of time evaluated. Increasing the percentage of ethanol in the solvent extraction (by using 70% ethanol) favored the TFC extraction for *Sambucus nigra* flowers HE. Conversely, Domínguez et al. observed an inverse correlation of the ethanolic ratio in extraction solvent and the TFC content from *Sambucus nigra* fruits HEs [68]. However, thus far, no reports have been made regarding the impact of the ethanol ratio in the extraction solvent on the HEs from *Sambucus nigra* flowers.

#### 3.1.5. The Optimization of the HEs

The optimization was aimed at the extraction conditions that led to the best extraction yields. This involved the application of constraints to mathematical models. Namely, the TPC and TFC values were maximized, and Modde software generated the working conditions to obtain the optimized HEs after the targets were set. The OHEs were prepared, and the obtained results are presented in Table 6 together with the theoretical values predicted by the software and the differences between them.

The TPC and TFC of the OHEs were very high, as established in QTPP of the HEs, to guarantee beneficial properties for wound-healing applications that will be further investigated. The TFC results were very close to the predicted values, confirming the validity of the model. On the other hand, the TPC results were found to be greater than the values predicted by the software. The complexity and inherent variability of the HE matrix may have contributed to the differences observed in the model. Despite these prediction limitations, the actual experimental results post-optimization exceeded the predicted values, even increasing the quality of the OHEs and underscoring the importance of experimental validation in conjunction with theoretical predictions.

### 3.2. Results of the Characterization of the OHEs

Identification and Quantification of Bioactive Compounds

Table 7 provides the qualitative and quantitative analysis of polyphenolic compounds, sterols, tocopherols, and procyanidins in the three OHEs, grouped according to their respective chemical classes.

Rutin (rutoside) was the major compound from the *Sambucus nigra* OHE (916.193 µg/mL), this result being in line with another research [69]. Chlorogenic acid (598.838 µg/mL), quercitrin (250.889 µg/mL), isoquercitrin (151.530 µg/mL), α-tocopherol (2273.811 ng/mL), campesterol (11.296 µg/mL), and stigmasterol (11.219 µg/mL) were also found in high concentrations in the *Sambucus nigra* OHE. The major constituents from the *Epilobium hirsutum* OHE were quercitrin (82.627 µg/mL) and β-sitosterol (159.665 µg/mL), and from the *Lythrum salicaria* OHE, isoquercitrin (10.830 µg/mL) and β-sitosterol (256.391 µg/mL). The implication of rutin in accelerating the wound-healing process was investigated in recent years. Based on appearance and histopathological assay after being tested on hyperglycemic rats, rutin promoted wound healing, reduced oxidative stress, inhibited the production of inflammatory cells, and affected angiogenesis by reducing vascular endothelial growth factor (VEGF) protein expression in the late stage of wound healing [70]. Chlorogenic acid (quercetin 3-O-rutinoside) is also considered to have a major impact on wound healing, owing to several studies of its properties. Moghadam et al. showed that chlorogenic acid induced an efficient wound closure on NHEKs (normal human epidermal keratinocytes) and had a high proliferative effect on NHDFs (normal human dermal fibroblasts). Moreover, chlorogenic acid exhibited pro-angiogenic activities on HUVECs (human umbilical vein endothelial cells) [71,72]. Chlorogenic acid has been proved to have anti-inflammatory activity by decreasing the secretion of TNF-α and IL-6 and having a re-epithelializing effect by the stimulation of L929 (mouse fibroblasts) cell growth [72]. The stimulation effect in epithelialization, angiogenesis, fibroblast proliferation, and collagen formation, together with the inhibition of polymorph nuclear leukocytes infiltration, were also demonstrated by Bagdas et al. and Chen et al. [73,74]. Together with each of these effects, the potent antioxidant activity by increasing superoxide dismutase, catalase, and glutathione, and decreasing lipid peroxidation, make chlorogenic acid a promising wound-healing agent [74]. Quercitrin (Quercetin-3-O-rhamnoside) has also been studied for its wound-healing properties. Gómez-Florit et al. have demonstrated that quercitrin promoted scarless wound healing in human gingival fibroblasts (HGFs), increased collagen IIIα1 and decorin levels, downregulated IL-6 messenger RNA levels, and decreased inflammatory mediator prostaglandin E2, the expression of profibrotic markers during wound healing, the MMP1/TIMP1 ratio (matrix metalloproteinase-1/Tissue Inhibitor of Metalloproteinases-1), and the ROS levels [75,76]. The efficacy of isoquercitrin was studied by Bhatia et al. after its incorporation in a cream for wound care. The study showed an increase in the percentage of wound contraction and a significant decrease in the period of epithelialization in isoquercitrin-based cream-treated groups compared with the control group, with a significant rise in Thiobarbituric Acid Reactive Substances (TBARS) and a decrease in reduced glutathione (GSH) levels [77].

Another component from the studied OHEs, α-tocopherol, is an important member of the tocopherols’ family and the biologically active variant of vitamin E in the mammalian body. α-tocopherol is known to exhibit high antioxidant and anti-inflammatory activity and to have promising potential to support the wound-healing process [78,79]. This bioactive compound may protect the polyunsaturated lipids and cell membrane against oxidative stress and regulate multiple cell signaling pathways [80]. Regarding its effect on wound healing, α-tocopherol may stimulate the polarization and migration of human keratinocytes through the protein kinase signaling cascade [81]. Also, it has been demonstrated that it may prevent wound infections with agents like methicillin-resistant *Staphylococcus aureus* (MRSA) by modulating the expression of connective tissue growth factors. Recently, some innovative topical systems were developed using α-tocopherol for accelerating the wound-healing process [80]. From the group of phytosterols, stigmasterol, campesterol, and, mostly, β-sitosterol have been found in our extracts. Stigmasterol can relieve oxidative stress inflammation and can inhibit cellular apoptosis [82], having also antifungal, antibacterial, and antioxidant potential [83]. Campesterol derivatives present biological potential as anti-inflammatory agents [84]. Moreover, β-sitosterol has been recently gaining interest in research [85] due to its antioxidant activity [86,87,88] and wound-healing effect [89] and for its analgesic, anti-inflammatory [90], and antimicrobial properties [91].

These OHEs contain a large variety of bioactive phytocompounds that can act on multiple pathways or phases in wound healing management. The phytochemical complex constituents can work together in synergy and complementarity through a multi-targeted approach, enhancing the therapeutic and protective effects for injured skin.

### 3.3. Results of Biological Activities Evaluation of the OHEs

#### 3.3.1. Results of the Antioxidant Activity of the OHEs

In Table 8, the results of the three assays for the antioxidant capacities of the OHEs and for the composed extract are presented (mean values ± SD, n = 3).

The most important antioxidant activities have been demonstrated for the *Lythrum salicaria* OHE (12.0192 TEs mg/mL OHE, 69.1414 TEs mM/OHE) and the *Epilobium hirsutum* OHE (156.8182 TEs mM/OHE). The *Epilobium hirsutum* OHE had the best antioxidant activity according to the TEAC assay and almost the same antioxidant activity as the *Lythrum salicaria* OHE through DPPH assay. The *Sambucus nigra* OHE showed a lower antioxidant potential. Phenolic acids and flavonoids are responsible for important antioxidant activity, deactivating free radicals based on their structural ability to donate hydrogen atoms to free radicals [64,92]. Tundis et al., who measured the antioxidant effects of ethanolic and methanolic HEs from the flowers and leaves of *Sambucus nigra*, also indicated an antioxidant activity through DPPH, FRAP, and ABTS assay [93]. Dawidowicz et al. showed that the scavenging potential of the DPPH radical by *Sambucus nigra* flowers or fruits HEs had higher antioxidant activities than that of the leaves HE [94]. Tunalier et al. also demonstrated good antioxidant activity of *Lythrum salicaria* HE, revealed by DPPH and FRAP assays. The aqueous methanolic extract was the best iron (III) reducer, with activity similar to that of butylated hydroxytoluene, the most potent DPPH scavenger [45]. A good antioxidant activity was demonstrated by Jamshidi et al., who concluded that in general, flower HEs showed better activity than leaf extracts and that the phenolic and flavonoid contents may be responsible for this property [95]. High antioxidant activity of *Epilobium hirsutum* HE was also observed by Kustova, using the same assays, supporting our findings [96]. Using DPPH assay, Karakaya et al. also observed an antioxidant effect of the *Epilobium hirsutum* HE [36]. High antioxidant activity was recorded by DPPH and TEAC assay after UTE in another study performed by Vlase et al. [24].

Antioxidant effects of herbal extracts in wound care are crucial [3]. It is well established that skin injuries are often linked to elevated oxidative species levels [12]. Specifically, reactive oxygen species (ROS) play a dual role in wound healing. On the one hand, they act as intracellular signaling mediators and are essential for immune defense against invading pathogens in dermal injuries. However, excessive ROS production leads to free radical generation and oxidation of cellular components, which can be detrimental. If ROS levels are not effectively neutralized, the wound-healing process may be delayed, resulting in chronic wounds, impaired tissue repair, or even neoplastic transformation [13,14]. Polyphenolic compounds, effective plant-derived antioxidants, have demonstrated their ability to neutralize ROS generated during inflammation while also mitigating the harmful effects of oxidative species on human tissues. By modulating the redox balance, they contribute to accelerating the wound-healing process [12,13,15,16,97].

#### 3.3.2. Results of the Antibacterial Activity of the OHEs

The results of the antibacterial activity of OHEs are presented in Table 9.

In the current study, the agar disk diffusion method revealed that all samples had a good inhibitory effect on both Gram-positive (*S. aureus* MSSA and *S. aureus* MRSA) and Gram-negative bacteria (*E. coli* and *P. aeruginosa*) compared with the negative control, represented by the solvent of the extracts (EtOH 70%) (*p* value < 0.0001) (Table 10). It can be observed that for all the studied bacterial strains, the most intense antibacterial activity was presented by the *Lythrum salicaria* OHE, followed by the *Epilobium hirsutum* OHE. Compared with the positive control, represented by gentamycin, the *Lythrum salicaria* OHE had approximately the same antibacterial effect as the positive control (non-significant difference) for *S. aureus* MSSA and MRSA. A weak inhibition was recorded with the *Sambucus nigra* OHE.

Aqueous HEs of *Sambucus nigra* flowers and leaves were previously investigated for their antibacterial properties but with no inhibitory effects observed on *E. coli* and *S. aureus* [98]. Conversely, another study demonstrated that lower concentrations of *Sambucus nigra* flowers aqueous HEs were needed to eradicate Gram-positive bacteria (*S. aureus*), but the HE showed a lesser effect on *P. aeruginosa* [99]. The HEs obtained from *Sambucus nigra* fruits were found to be very effective on *S. aureus, P. aeruginosa* [100], and *E. coli* [101]. In another study, it was established that the *Sambucus nigra* flowers HEs were the most toxic to all the bacteria tested, including MRSA, compared with the other HEs [102]. In prior work, a hydro-methanolic HE from *Lythrum salicaria* aerial parts demonstrated a clear antibacterial activity against *E. coli* and a slight antibacterial activity against *S. aureus* [103]. Another study reported that methanolic HE of *Lythrum salicaria* aerial parts showed antibacterial effects on *S. aureus* but no effects on *E. coli* or *P. aeruginosa* [104]. However, another study reported that ethanolic HE of *Lythrum salicaria* aerial parts had antibacterial activity on *S. aureus, E. coli*, and *P. aureus*, supporting our results [44,105]. The HE’s efficacy on multi-drug-resistant *P. aeruginosa* was also demonstrated by another study [44,106]. The antibacterial effects of ethanolic HEs from the aerial parts of *Epilobium hirsutum* were previously reported. The results showed that the HE had antibacterial effects on *S. aureus* and *P. aeruginosa*, in alignment with our findings [107]. Thus, the hydroethanolic HEs from these species have not been widely examined for their antibacterial activity on these bacterial strains, and the results are very different from one study to another, so our findings broadened the current knowledge in this field. The quantitative antimicrobial potential using the MIC method was tested, and the MIC values ranged from 6.25 to 50 mg/mL (Table 10).

The MIC values for antibiotics typically fall between 0.01 and 10 µg/mL, while herbal extracts are regarded as antimicrobials when their MICs range from 100 to 1000 µg/mL. Some researchers even suggest varying thresholds based on the specific compound. Varying thresholds based on the specific compound were reported, making it difficult to obtain comparable and reliable results [108].

Of all the extracts, the *Sambucus nigra* OHE exhibited the highest MIC value against *S. aureus* MRSA (>25 mg/mL). A MIC value of 25 mg/mL was exhibited by *Sambucus nigra* OHE against *S. aureus* MSSA, *E. coli*, and *P. aeruginosa* and by the *Epilobium hirsutum* OHE against *P. aeruginosa.* Lower MIC values, of 12.5 mg/mL, were observed for the *Epilobium hirsutum* OHE against both *Staphylococcus* strains and for the *Lythrum salicaria* OHE against *P. aeruginosa.* The lowest MIC values (6.25 mg/mL) were recorded for the *Epilobium hirsutum* OHE against *E. coli*, and for the *Lythrum salicaria* OHE against both *Staphylococcus* strains.

Antibacterial effects of wound care products are essential, as one of the main causes of morbidity and mortality worldwide is bacterial infection of wounds. This can lead to various complications, such as abscess formation and even sepsis in severe cases. Wound infections can also delay the healing process of wounds and cause chronic or impaired healing [3,18,19]. The bacterial strains chosen for the evaluation of antibacterial activity are among those found most often in wound infections. It has been noted that *S. aureus* is the bacterium most often isolated from various wound types, and a significant portion of *S. aureus* are methicillin-resistant *S. aureus* (MRSA). Furthermore, anaerobes like *P. aeruginosa* are commonly found in wounds from burns and surgical incisions, whereas facultatively anaerobic bacteria like *E. coli* are commonly found in chronic wounds [18]. It was previously documented that the polyphenols and phenolic acids contained in our extracts, like quercetin and chlorogenic, p-coumaric, caffeic, ferulic, gallic, vanillic, and protocatechuic acids, may be responsible for inhibiting the growth of *S. aureus, E. coli*, and *P. aeruginosa* bacteria [99,101,103].

#### 3.3.3. Results of the In Vitro Cell Culture Assays for the OHEs

##### Results of the Cell Viability

The results of the cell viability assay are presented in Table 11. The biocompatible concentrations of HEs were further used for the evaluation of antioxidant and anti-inflammatory activities, and the highest non-toxic (biocompatible) concentrations for OHEs were used for the wound-healing assay.

For the *Sambucus nigra* OHE, no cytotoxic effects were observed. To the best of our knowledge, the cell viability of *Sambucus nigra* flowers HEs on HaCaT was not previously reported; only the extracts of fruits and leaves have been evaluated so far. The biocompatibility of *Sambucus nigra* leaves HE (5–100 µg/mL) was demonstrated by Skowronska et al. on HaCaT and NHDFs (normal human dermal fibroblasts) [27]. In a study conducted by Pereira et al., *Sambucus nigra* flowers HE showed biocompatibility with human fibroblast cells (MRC-5) used to compare the activity in cancer cells, showing selectivity [69]. Wójciak et al. observed that the *Sambucus nigra* fruits HEs could stimulate the activity of keratinocytes, but in the case of fibroblasts, they observed a slight inhibition of cell viability after exposure to two higher concentrations (250 and 1000 µg/mL) [30]. Pei Lin et al. also demonstrated that cell proliferation was improved by *Sambucus nigra* fruits HEs in UVB-irradiated HaCaT [109]. Concerning fibroblast viability, some other papers are in accordance with our findings. Filip et al. reported that *Sambucus nigra* fruit HE is biocompatible with human gingival fibroblasts, even at high doses (50–100 μg polyphenols (GAE)/mL). After including the extract in silver nanoparticles, it was observed that viability increased in a dose-dependent manner up to the dose of 50 μg/mL [110]. An increase in the proliferation of human skin fibroblast Hs27 cells was also observed by Studzinska-Sroka et al. after treatment with some *Sambucus nigra* leaves HE [31]. On the other hand, for *Lythrum salicaria* and *Epilobium hirsutum* HEs, a dose-dependent increase in cytotoxicity was observed. The *Epilobium hirsutum* HE was also evaluated for anticancer activity by Vlase et al., showing a dose-dependent toxicity on BJs [24].

##### Results of the Antioxidant Activity in Cell Cultures

**Figure 2:**
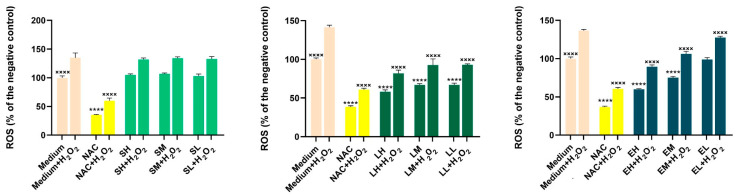
Antioxidant effects of the three OHEs on HaCaT. The data are expressed as relative means ± standard deviation of three biological replicates, each one including six technical replicates. The values were expressed as relative values compared with the negative control (DMSO 0.2%) (100%). Asterisks (*—compared with negative control, x—compared with medium + H_2_O_2_) indicate statistically significant differences in comparison with positive control (ANOVA test), where ****/^xxxx^ = *p* < 0.0001. S—*Sambucus nigra* OHE, L—*Lythrum salicaria* OHE, E—*Epilobium hirsutum* OHE, H, M, L = high, medium, low biocompatible concentrations of each OHE (Table 10).

**Figure 3:**
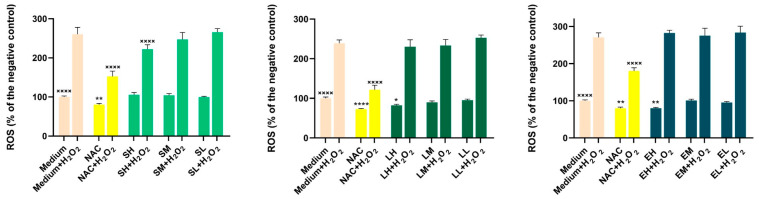
Antioxidant effects of OHEs on BJ. The data are expressed as relative means ± standard deviation of three biological replicates, each one including six technical replicates. The values were expressed as relative values compared with the negative control (DMSO 0.2%) (100%). Asterisks (*—compared with negative control, x—compared with medium + H_2_O_2_) indicate statistically significant differences in comparison with positive control (ANOVA test), where ****/^xxxx^ = *p* < 0.0001, ** = *p* < 0.01, * = *p* < 0.05, NAC—N-acetylcysteine, S—*Sambucus nigra* OHE, L—*Lythrum salicaria* OHE, E—*Epilobium hirsutum* OHE, H, M, L = high, medium, low non-toxic concentrations of each OHE (Table 11).

The results of antioxidant activity in cell cultures are presented in Figure 2 and Figure 3.

To assess the potential antioxidant effects of the extracts, three concentrations that did not affect the cellular viability were selected (Table 11). In the HaCaT cell line, exposure to the *Lythrum salicaria* OHE alone at all three concentrations (H-150, M-75, L-25 µg/mL) and to the *Epilobium hirsutum* OHE alone at the two higher concentrations (H-50, M-25 µg/mL) significantly lowered the basal oxidative status compared with the negative control (*p* < 0.0001). Similarly, N-acetylcysteine (NAC) treatment significantly decreased ROS levels under non-stimulated conditions (*p* < 0.0001), while the *Sambucus nigra* OHE showed no effect on ROS production in this state. In the BJ cell line, under non-stimulated conditions, only the highest concentrations of the *Lythrum salicaria* (H-50 µg/mL) and *Epilobium hirsutum* (H-25 µg/mL) OHEs produced a significant reduction in basal oxidative status (*p* < 0.01). Exposure to H_2_O_2_ alone caused a statistically significant increase in ROS levels relative to the negative control. However, pre-incubation with the *Lythrum salicaria* OHE (H-150, M-75, L-25 µg/mL) and the *Epilobium hirsutum* OHEs (H-50, M-25, L-1 µg/mL) at all three concentrations partially inhibited ROS formation in the HaCaT cell line (*p* < 0.0001).

Compared with HaCaT, the BJ cell line was less responsive at the extracts’ pre-treatment; only the *Sambucus nigra* OHE, at the highest concentration tested (H-400 µg/mL), was able to reduce the ROS formation in a significant manner (*p* < 0.0001). As expected, under stimulated conditions, NAC treatment significantly reduced ROS levels in both cell lines (*p* < 0.0001). The results are in agreement with the colorimetric antioxidant assay results, emphasizing the antioxidant activity of the *Lythrum salicaria* and *Epilobium hirsutum* OHEs. Our results are in accordance with other research, which demonstrated the capacity of *Epilobium hirsutum* extracts to reduce the production of ROS from f-MLP (formyl-met-leuphenylalanine) and PMA (4beta-phorbol-12beta-myristate-alpha13-acetate)-induced neutrophils [40]. For *Sambucus nigra* leaves HE, Skowronska et al. observed a dose-dependent inhibition in the production of ROS by human neutrophils stimulated with the bacteria-derived peptide (f-MLP). A dose-dependent scavenging activity of superoxide anion and nitric oxide and a H_2_O_2_ scavenging activity for ethanolic HEs, compared with aqueous extracts, were also observed [111]. The antioxidant effect of the *Lythrum salicaria* HE was studied by Piwowarski and Kiss, showing inhibition of f-MLP- and PMA-induced ROS production [43].

##### Results of the Anti-Inflammatory Activity in Cell Cultures

**Figure 4:**
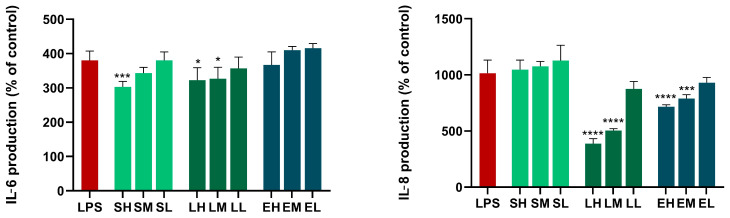
The effects of the OHEs on IL-6 and IL-8 production. Data are expressed as mean ± SD (n ≥ 3). Asterisks (*) indicate statistically significant differences in comparison with positive control (ANOVA test, where **** = *p* < 0.0001, *** = *p* < 0.001, * = *p* < 0.05), LPS—lipopolysaccharide, S—*Sambucus nigra* OHE, L—*Lythrum salicaria* OHE, E—*Epilobium hirsutum* OHE, H, M, L = high, medium, low non-toxic concentrations of each OHE (Table 11).

The results depicted in Figure 4 show that the *Sambucus nigra* OHE (H-400 µg/mL) inhibited IL-6 secretion from LPS-stimulated BJs. For the *Lythrum salicaria* OHE, the two highest concentrations tested (H-50, M-25 µg/mL) exhibited a statistically significant inhibition of IL-6 production, while in the case of the *Epilobium hirsutum* OHE, no effect was observed in the LPS-stimulated BJs. In the case of IL-8 production, the *Epilobium hirsutum* OHE (H-25, M-10, L-1 µg/mL) and the *Lythrum salicaria* OHE (H-50, M-25, L-1 µg/mL) inhibited the secretion of IL-8, each at the two highest concentrations tested. To the best of our knowledge, this is the first research that studied the anti-inflammatory effects of *Sambucus nigra* flower and *Lythrum salicaria* HEs on BJs. The inhibition of IL-6 secretion by *Sambucus nigra* flowers HE (1, 10, 100 µg/mL) was previously demonstrated on LPS-stimulated neutrophils and macrophages, correlated with the same anti-inflammatory effects as rutin, the major compound of the HE [34]. Conversely, the secretion of IL-8 from neutrophils was previously found to be increased or not influenced by *Sambucus nigra* leaves HEs [111]. Wójciak et al. have reported that extracts of *Sambucus nigra* leaves exhibited a concentration-dependent inhibition of IL-6 secretion from LPS-stimulated fibroblasts. Their research also showed that the protocatechuic acid, a phenolic acid found in the composition of the extract, suppressed the levels of IL-6 [30]. Other research demonstrated that *Sambucus nigra* fruit HEs suppressed mRNA expression of IL-6 and, consequently, the secretion of IL-6 in RAW 264.7 macrophages [112]. Skowronska et al. reported that *Sambucus nigra* leaves HEs inhibit TNF-α secretion by LPS-stimulated human neutrophils, but no effect or increase in the IL-8 levels was observed. They also demonstrated an inhibition of the activity of lipoxygenase, confirming the anti-inflammatory effects by multiple mechanisms [111]. In another study, they demonstrated that UVB-irradiated HaCaT cells treated with the *Sambucus nigra* leaves HEs increased IL-6 levels but decreased IL-8 levels. Regarding TNF-α and IFN-γ stimulation of HaCaT cells, *Sambucus nigra* leaves HE increased IL-6 secretion and slightly decreased IL-8 secretion. Concerning lipoteichoic acid-stimulated fibroblasts (NHDFs), their findings showed a decrease in IL-8 and IL-6 release [27]. The inhibition of IL-6 and IL-8 release from BJs after treatment with *Epilobium hirsutum* HE was previously investigated by Vlase et al., with promising results [24]. Decreasing the gene expression of IL-8 was also demonstrated by Ak et al. in human prostate cancer PC3 cells [41]. Other research demonstrated that the *Epilobium hirsutum* HEs exhibited anti-inflammatory effects by other mechanisms, namely, by the inhibition of myeloperoxidase release from stimulated neutrophils [40], and that they reduced the gene expression of COX-2 and TNF-α in human prostate cancer PC3 cells [41]. For *Lythrum salicaria* HE, a scarce amount of data exists regarding its anti-inflammatory effects. In the study performed by Piwowarski and Kiss, the aqueous extract was shown to have a moderate inhibitory effect toward the lipopolysaccharide (LPS)-triggered production of IL-8 by neutrophils, possibly due to ellagitannins [43].

##### Results of the Measurement of Nitric Oxide (NO) Production

**Figure 5:**
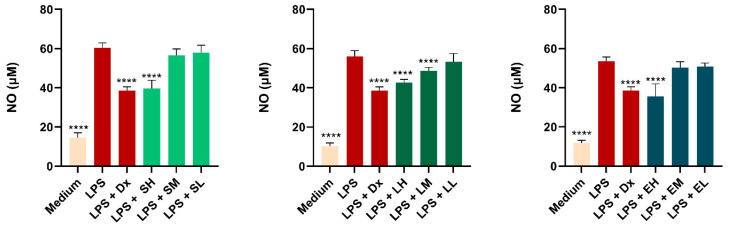
The effects of the OHEs on NO production. Data are expressed as mean ± SD (n ≥ 3). Asterisks (*) indicate statistically significant differences in comparison with positive control (ANOVA test), where **** = *p* < 0.0001), LPS—lipopolysaccharide, Dx—dexamethasone, S—*Sambucus nigra* OHE, L—*Lythrum salicaria* OHE, E—*Epilobium hirsutum* OHE, H, M, L = high, medium, low non-toxic concentrations of each OHE (Table 11).

Macrophages play important roles in inflammation through the production of several pro-inflammatory molecules, including NO. To measure the inhibitory effect of plant extracts on pro-inflammatory mediator production, NO levels were investigated using RAW 264.7 cells stimulated by LPS. The results depicted in Figure 5 show that the nitrite accumulation in the cells increased following the LPS treatment in a statistically significant manner compared with cells exposed to cell medium. Treatment with *Sambucus nigra* and *Epilobium hirsutum* OHEs, each at the highest concentrations tested, achieved a statistically significant decrease in NO production in cells stimulated by LPS, comparable with the one observed after dexamethasone exposure. In the case of the *Lythrum salicaria* OHE, effects were noticed for the two higher concentrations in a dose-dependent manner (*p* < 0.005).

In a previous work, Ferreira et al. tested the anti-inflammatory effects of extracts from three cultivars of *Sambucus nigra* HEs from fruits (Sabugueiro, Sabugueira, and Bastardeira) in concentrations up to 100 μg/mL. The results showed a significant decrease in NO production: Sabugueira reduced NO production by approximately 75%, Sabugueiro reduced NO production by 69.56%, and Bastardeira reduced NO production by 41.22%. In our case, the *Sambucus nigra* flower HE at 400 ug/mL, the highest concentration tested, led to a NO scavenging rate of 34.35%, not so high as the three extracts used by Ferreira, but comparable to the effects observed in the case of the Bastardeira cultivar. The difference in the results could be explained by the fact that fruit extracts, due to their concentrated polyphenol and antioxidant content, may often have a higher overall anti-inflammatory effect compared with flower extracts [113]. On the other hand, in vivo studies conducted on an animal model of rhinosinusitis confirmed the anti-inflammatory potential of *Sambucus nigra* HE after oral administration [29]. Kim et al. investigated water HE from *Lythrum salicaria* leaves and reported a NO scavenging rate of 46.9%. In our study, conducted on the aerial parts of the plant, the highest extract concentration showed a NO scavenging rate of 24%. This significant difference suggests that the leaf extract has a stronger anti-inflammatory effect than the extract from the aerial parts, indicating variability in the chemical composition or effectiveness of different parts of the plant in reducing LPS-induced NO production [114]. Regarding the *Epilobium hirsutum* HE, our study confirms the ability of *Epilobium* sp. HE to reduce NO secretion in RAW 264.7 cells, as observed in the literature at concentrations of 1000 and 100 µg/mL, further validating its capacity to modulate oxidative stress [115].

##### Results of the Wound Healing Assay

**Figure 6:**
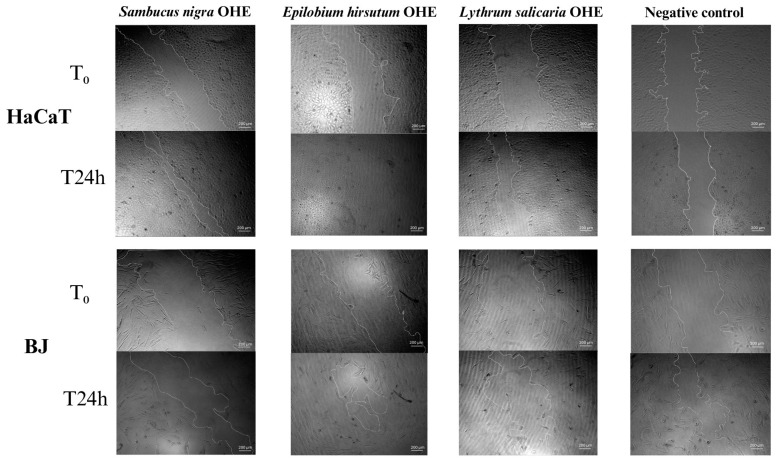
Graphical representation of wound-healing properties of the OHEs after scratch assay on HaCaT and BJ incubated for 24 h.

In Table 12, the results concerning the wound-healing activity of the three optimized HEs are presented in the highest biocompatible concentration from Table 10. Thus, the wound closure and cell migration were calculated after 24 h. Moreover, Figure 6 presents the wound-healing properties of the assessed HEs after scratch assay performed on HaCaT and BJ cell lines incubated for 24 h.

The most effective wound-healing effect on HaCaT and BJ after 24 h was found for the *Epilobium hirsutum* OHE (H-50 µg/mL in HaCaT and H-25 µg/mL in BJ), having significantly higher wound closure (*p* < 0.001) and cell migration (*p* < 0.05) values compared with the negative control. Thus, the *Epilobium hirsutum* OHE determined almost the total closure of the wound in 24 h. A significant wound-healing effect was also observed for the *Sambucus nigra* OHE in HaCaT (*p* < 0.05). The results for the *Sambucus nigra* flowers OHE are similar to those obtained by Skowronska et al. for *Sambucus nigra* leaves HE with 70% ethanol on HaCaT cell lines (wound closure 50.03 ± 4.05% and negative control 23.74 ± 7.32%) [13]. Another study made by Studzinska-Sroka evaluated the wound-healing properties of *Sambucus nigra* leaves HE on the human skin fibroblasts Hs27. It was shown that the most active HE that was studied closed the wound by 64.4 ± 3.7% (24 h) and 79.0 ± 3.0% (36 h) [17]. The wound-healing effects of *Lythrum salicaria* HE were assessed in second-degree burn wounds in rats when the wound contraction percentage was 89.5 ± 3.7%, and a well-organized epidermal layer and normal appearance in the dermis layer were observed [46]. A *Lythrum salicaria* HE was previously analyzed on normal human epidermal keratinocytes (NHEKs), reconstructed human epidermis, and full-thickness reconstructed skin to determine the effects from gene expression to skin morphology. It was shown that HE helped in the structuration of the cornified layer and promoted the production of proteins, allowing for proper and efficient epidermis functions and the differentiation of its constitutive cells [47].

### 3.4. Study Limitations

A potential limitation of this study is the absence of advanced biological assays that could further elucidate the precise mechanisms involved in wound healing, such as matrix metalloproteinase (MMP) activity related to collagen remodeling (e.g., MMP-1 and MMP-9), protease activity, growth factor levels (e.g., platelet-derived growth factor—PDGF), stem/progenitor cell recruitment (SPCs), microRNA (miRNA) expression, and C-X-C motif chemokine ligand 6 (CXCL-6) levels.

However, the primary objective of this study was to establish the bioactive potential of the OHEs through comprehensive antioxidant, antibacterial, and anti-inflammatory assessments, serving as an essential preliminary step in understanding their broader applicability. Given the complexity of wound healing and the multitude of contributing factors, we focused on evaluating cellular viability and cytokine-mediated anti-inflammatory effects, which are particularly relevant in the early stages of the wound-healing process.

While further studies are warranted to explore additional mechanisms and validate the therapeutic potential of these extracts in wound healing, our findings provide valuable insights into their biological activity. Considering the limited existing data on the bioactive properties of the tested extracts, this study makes a significant contribution by demonstrating their antioxidant, antibacterial, and anti-inflammatory effects alongside potential wound-healing-promoting activities, as evidenced by the scratch assay.

## 4. Conclusions

There is limited scientific research on the application of *Lythrum salicaria* HE, *Epilobium hirsutum* aerial parts HE, and *Sambucus nigra* flower HE in wound care, despite their use in traditional medicine. Using the QbD method provided an important advantage in developing high-quality extracts and understanding the influence of the extraction parameters on the phytocompounds’ extraction. The optimal extraction conditions to reach the maximization of total polyphenol (TPC) and flavonoid (TFC) contents were obtained for each analyzed plant material: UTE with 70% ethanol for 3 min for *Epilobium hirsutum* and *Lythrum salicaria*, and 6 min for *Sambucus nigra*. Rutin was the major compound from the *Sambucus nigra* OHE. Chlorogenic acid, quercitrin, isoquerictrin, α-tocopherol, campesterol, and stigmasterol were also found in high concentrations in the *Sambucus nigra* OHE. The major constituents from the *Epilobium hirsutum* OHE were quercitrin and β-sitosterol, and those from the *Lythrum salicaria* OHE were isoquecitrin and β-sitosterol. For all the studied bacterial strains (*Escherichia coli, Staphylococcus aureus* MSSA, *Staphylococcus aureus* MRSA, and *Pseudomonas aeruginosa*), all the OHEs presented statistically significant differences compared with the negative control. Compared with the positive control, represented by gentamycin, the *Lythrum salicaria* OHE had approximately the same antibacterial effect as the positive control (non-significant difference) for *S. aureus* MSSA and MRSA.

Regarding the in vitro evaluations on both HaCaT and BJ, the *Sambucus nigra* OHE displayed an increase in cell viability. The *Lythrum salicaria* and *Epilobium hirsutum* OHEs showed important antioxidant capacity in the HaCaT cell line, significantly lowering the basal oxidative status compared with the negative control.

The *Sambucus nigra* and *Lythrum salicaria* OHEs showed anti-inflammatory effects by significantly decreasing IL-6 and IL-8 levels. The *Sambucus nigra* and *Epilobium hirsutum* OHEs at the highest concentrations tested significantly decreased NO production in macrophages stimulated by LPS, comparable with that observed after dexamethasone exposure, while in the case of the *Lythrum salicaria* OHE, effects were noticed for the two higher concentrations in a dose-dependent manner. The wound closure and cell migration were significantly increased, especially in HaCaT, for the *Epilobium hirsutum* and *Sambucus nigra* OHEs.

Thus, these OHEs showed great potential for future uses in various types of wounds and phases of the physiological wound-healing process due to the combination of anti-inflammatory, antimicrobial, antioxidant, and wound-healing activities. These effects could be crucial in treating infected wounds, in overcoming the inflammatory phase, and in mitigating oxidative stress-related damage, thus potentially reducing the risk of delayed healing and chronicization. In addition, by promoting tissue regeneration, they could efficiently aid in the proliferative phase of wound healing. By comparing the composition and biological effects of these OHEs, we observed that their properties may be synergistic and complementary, suggesting the potential for combining the three OHEs in future topical systems for wound care. More research is needed for in vivo demonstrations of these effects to further develop formulations that can use the full potential of these herbal phytochemical complexes to accelerate the overall healing process. This study could serve as a valuable reference for future research, providing scientific validation for the traditional use of these widely distributed plants from European wild flora in wound care by investigating their biological effects.

## Figures and Tables

**Table 1:** Details regarding the harvesting period and location of the plant materials and their voucher specimen number.

Plant Material	Voucher Specimen No.	Harvest Period and Location
*Sambucus nigra* flowers	139.1.2.1/05.2022	May 2022, Stremț, Alba County
*Lythrum salicaria* aerial parts	60.3.1.1/06.2022	June 2022, Săndulești, Cluj County
*Epilobium hirsutum* aerial parts	62.5.1.1/07.2022	July 2022, Săndulești, Cluj County

**Table 2:** Input and output variables of the DoEs for each plant material.

Input Variables and Variation Levels (Independent Variables, Factors)			Output Variables (Dependent Variables, Responses)
Qualitative variable	Extraction method—X1	UTE	TPC—Y1 μg GAE/mL HE
		USE	
Quantitative variable	Extraction time—X2	3, 5, 10 min	TFC—Y2 μM QAE/HE
	Ethanol ratio in the extraction solvent—X3	30%, 50%, 70%	

UTE—ultra-turrax-assisted extraction, USE—ultrasound-assisted extraction, TPC—total polyphenol content, expressed as μg gallic acid equivalent (GAE)/mL HE; TFC; total flavonoid content, expressed as μM quercetin equivalents (QAE)/HE.

**Table 3:** Overview of quality target profile of the OHEs for wound-healing properties.

Parameter	Justification [3,31,63,64]	Target
Antioxidant capacity	To regulate the redox environment from the wound to combat oxidative damage, which can otherwise hinder the healing process, leading to delayed healing or chronic wounds.	Maximize
Antibacterial activity	To prevent or treat the infections caused by the most common bacteria in wounds, like *Staphylococcus aureus* and resistant strains (e.g., MRSA, methicillin-resistant *Staphylococcus aureus*), *Escherichia coli* (frequently met in chronic wounds), *Pseudomonas aeruginosa* (commonly isolated from wounds following surgeries and burns).	
Anti-inflammatory activity	To reduce excessive inflammation, minimize tissue damage, decrease the associated pain, overcome the inflammatory phase of the physiological process of wound healing, and to prevent the chronicity of the wound and scar formation.	
Cell viability	To ensure the biocompatibility with implied cell lines (HaCaT, BJ), increase fibroblast and keratinocytes proliferation, and stimulate the growth factors involved in the process of healing (e.g., FGF—fibroblast growth factor, EGF—epidermal growth factor).	
Wound-healing activity	To stimulate the growth factors involved in the process of healing and the migration of the cells and to hasten the wound closure.	
All the above may be enhanced by increasing the content of polyphenols and flavonoids.		
Total polyphenol content (TPC)	To exert the antioxidant, antibacterial, anti-inflammatory, and wound-healing activities.	Maximize
Total flavonoid content (TFC)	To exert the antioxidant, antibacterial, anti-inflammatory, and wound-healing activities.	

**Table 4:** Statistical parameters for ANOVA test and quality of fit.

HEs	Response	R^2^	Q^2^	*p*-Value	Lack of Fit	Model Validity	Reproducibility
*Sambucus* *nigra*	Y1-TPC	0.562	−0.797	0.386	0.001	−0.200	0.999
	Y2-TFC	0.672	0.430	0.004	0.165	0.549	0.913
*Lythrum* *salicaria*	Y1-TPC	0.698	0.418	0.010	0.285	0.685	0.835
	Y2-TFC	0.589	0.335	0.012	0.108	0.443	0.931
*Epilobium* *hirsutum*	Y1-TPC	0.917	0.690	0.000	0.440	0.795	0.912
	Y2-TFC	0.870	0.794	0.000	0.124	0.477	0.975

Y1-TPC, total polyphenolic content, expressed as μg gallic acid equivalent (GAE)/mL HE; Y2-TFC, total flavonoid content, expressed as μM quercetin equivalent (QAE)/HE.

**Table 5:** DoE matrix.

Exp. No.	Input Variables			Obtained Output Variables					
	All HEs			*Sambucus nigra* HE		*Lythrum salicaria* HE		*Epilobium hirsutum* HE	
	X1	X2	X3	Y1-TPC	Y2-TFC	Y1-TPC	Y2-TFC	Y1-TPC	Y2-TFC
N1	UTE	3	30	4889.61 ± 195.58	2296.96 ± 45.93	6591.62 ± 131.83	638.97 ± 25.55	6530.52 ± 195.91	1554.6 ± 31.09
N2	USE	3	30	4749.95 ± 427.49	1032.13 ± 20.64	6059.20 ± 121.18	328.26 ± 29.54	5587.87 ± 167.63	729.71 ± 14.59
N3	UTE	10	30	5282.38 ± 369.76	1969.75 ± 39.39	6757.46 ± 405.44	471.24 ± 9.42	6705.09 ± 536.41	1714.08 ± 68.56
N4	USE	10	30	4802.32 ± 240.11	1897.16 ± 56.91	6094.11 ± 243.76	306.27 ± 18.37	5552.96 ± 166.58	823.19 ± 57.62
N5	USE	5	30	5230.01 ± 104.60	1695.89 ± 33.91	5344.48 ± 213.77	157.79 ± 4.73	5413.31 ± 108.26	677.46 ± 20.32
N6	UTE	3	70	4976.89 ± 447.92	2481.18 ± 173.68	7158.96 ± 143.17	773.7 ± 38.68	6582.89 ± 65.82	1906.55 ± 38.13
N7	USE	3	70	5718.79 ± 285.93	2663.76 ± 159.82	6478.15 ± 583.03	1023.92 ± 40.95	5491.86 ± 439.34	1131.16 ± 56.55
N8	UTE	10	70	5046.72 ± 252.33	3642.62 ± 218.55	6713.82 ± 268.55	913.93 ± 36.55	5692.61 ± 170.77	2068.78 ± 186.19
N9	USE	10	70	4907.07 ± 196.28	2143.53 ± 150.04	5989.37 ± 59.89	473.99 ± 23.69	5291.11 ± 211.64	759.95 ± 68.39
N10	UTE	5	50	4959.44 ± 297.56	2931.02 ± 205.17	7246.25 ± 434.77	575.733 ± 34.54	6635.27 ± 597.17	1606.84 ± 16.06
N11	USE	5	50	4872.15 ± 389.77	1778.47 ± 124.49	5178.39 ± 268.91	148.78 ± 1.48	5238.74 ± 52.38	612.23 ± 24.48
N12	USE	5	50	4854.70 ± 97.09	1921.36 ± 115.28	5665.67 ± 54.65	185.28 ± 11.12	5247.47 ± 367.32	522.73 ± 47.04
N13	USE	5	50	4872.15 ± 146.16	2125.85 ± 148.81	5422.04 ± 271.10	210.78 ± 6.32	5509.32 ± 220.37	575.73 ± 34.54

UTE—ultra-turrax-assisted extraction; USE—ultrasound-assisted extraction; X1—extraction method; X2—extraction time; X3—ethanol ratio in the extraction solvent; Y1-TPC, total polyphenolic content, expressed as μg gallic acid equivalent (GAE)/mL HE; Y2-TFC, total flavonoid content (TFC), expressed as μM quercetin equivalent (QAE)/HE.

**Table 6:** The variation factors for obtaining the OHEs and the results of their analysis.

	**Extraction conditions for obtaining the OHEs**					
Variation factors	*Sambucus nigra*		*Lythrum salicaria*		*Epilobium hirsutum*	
X1	UTE		UTE		UTE	
X2 (min)	6		3		3	
X3 (% EtOH)	70		70		70	
	**Results of analysis of the OHEs**					
	*Sambucus nigra*		*Lythrum salicaria*		*Epilobium hirsutum*	
	TPC	TFC	TPC	TFC	TPC	TFC
Predicted maximal values	5459.17	3208.05	7067.61	923.85	6628.25	1925.44
Experimental values	5750.01 ± 173.21	3030.39 ± 42.21	7653.11 ± 974.226	1025.74 ± 39.01	6950.35 ± 790.811	1973.03 ± 21.31

UTE—ultra-turrax-assisted extraction; Y1-TPC, total polyphenolic content, expressed as μg gallic acid equivalent (GAE)/mL OHE; Y2-TFC, total flavonoid content, expressed as μM quercetin equivalent (QAE)/OHE.

**Table 7:** Identification and quantification of bioactive compounds from the OHEs.

Bioactive Compounds		*Sambucus nigra*	*Epilobium hirsutum*	*Lythrum salicaria*
Phenolic acids (µg/mL)	Caftaric acid	-	<LOQ	-
	Chlorogenic acid	598.838 ± 35.930	<LOQ	3.422 ± 0.239
	4-O-caffeoylquinic acid	40.811 ± 2.856	-	-
	*p*-coumaric acid	<LOQ	<LOQ	-
	Gentisic acid	<LOQ	-	-
	Gallic acid	2.199 ± 0.087	36.827 ± 1.104	29.366 ± 2.348
	Protocatechuic acid	8.395 ± 0.755	-	0.167 ± 0.009
	Vanillic acid	0.155 ± 0.012	-	-
Flavanols (µg/mL)	(+)-Epicatechin	0.331 ± 0.003	0.081 ± 0.001	0.082 ± 0.002
	(-)-Catechin	0.042 ± 0.001	0.213 ± 0.008	0.022 ± 0.001
	Epigallocatechin	0.415 ± 0.004	1.147 ± 0.068	0.126 ± 0.009
	Epigallocatechin gallate	-	0.538 ± 0.021	-
	Procyanidin A1	0.216 ± 0.014	0.067 ± 0.004	-
	Procyanidin B1	0.138 ± 0.010	-	-
	Procyanidin B2	0.321 ± 0.006	0.113 ± 0.009	0.147 ± 0.007
Flavonols (µg/mL)	Hyperoside	-	33.160 ± 1.658	0.507 ± 0.045
	Isoquercitrin	151.530 ± 10.607	5.744 ± 0.344	10.830 ± 0.758
	Rutin	916.193 ± 27.485	1.302 ± 0.091	<LOQ
	Myricetin	-	22.618 ± 0.227	-
	Quercitrin	250.889 ± 12.544	82.627 ± 2.478	<LOQ
	Quercetol	1.935 ± 0.135	<LOQ	-
	Kaempferol-3-Rhamnoside	-	5.943 ± 0.118	-
Flavones (µg/mL)	Luteolin	88.966 ± 5.338	-	1.781 ± 0.053
	Apigenin	-	-	0.285 ± 0.014
Sterols (μg/mL)	Ergosterol	0.441 ± 0.022	-	-
	Stigmasterol	11.219 ± 0.561	-	2.714 ± 0.108
	Beta-Sitosterol	417.593 ± 12.527	638.215 ± 51.057	256.391 ± 12.819
	Campesterol	11.296 ± 0.225	1.883 ± 0.094	1.104 ± 0.066
	Brassicasterol	1.163 ± 0.034	3.210 ± 0.096	0.845 ± 0.059
Tocopherols (ng/mL)	α-tocopherol	2273.811 ± 181.904	290.202 ± 17.412	837.842 ± 50.268
	δ-tocopherol	29.419 ± 0.295	159.665 ± 4.788	8.212 ± 0.656
	γ-tocopherol	202.909 ± 2.031	847.473 ± 25.422	103.864 ± 2.076

<LOQ—below the quantification limit of the analytical method (compounds identified based on MS spectra but not quantified).

**Table 8:** The antioxidant capacities of the OHEs.

OHEs	DPPH Assay	FRAP Assay	TEAC Assay
*Sambucus nigra*	7.5394 ± 0.3982	29.5620 ± 1.0730	48.8131 ± 15.6024
*Lythrum salicaria*	12.0192 ± 0.9553	69.1414 ± 9.0016	65.8586 ± 4.9098
*Epilobium hirsutum*	11.6666 ± 0.5266	21.5528 ± 5.1290	156.8182 ± 0.9185

DPPH assay—results expressed as Trolox Eq (TEs) mg/mL OHE; FRAP assay—results expressed as Trolox Eq (TEs) mM/OHE; TEAC assay—Trolox Eq (TEs) mM/OHE.

**Table 9:** Antibacterial activity of the OHEs.

Bacterial Strains	*Sambucus nigra*	*Lythrum salicaria*	*Epilobium hirsutum*	NC	PC
	Zone of Inhibition (mm)				
*Escherichia coli*, ATCC 25922	9.67 ± 0.58 ****	16.33 ± 1.15 **	20.00 ± 0.00 *	0	18.5 ± 0.00
*Staphylococcus aureus* MRSA, ATCC 700699	8.33 ± 0.58 ****	20.17 ± 0.29	18.00 ± 1.00 **	0	20.0 ± 0.00
*Staphylococcus aureus* MSSA, ATCC 25923	9.00 ± 1.00 ****	20.33 ± 0.57	18.00 ± 0.50 **	0	20.0 ± 0.00
*Pseudomonas aeruginosa*, ATCC 27853	9.33 ± 1.15 ****	15.00 ± 1.73 *	15.67 ± 1.52 *	0	18.5 ± 0.00

NC = negative control (solvent—EtOH 70%), PC = positive control (gentamycin). Data are expressed as mean ± SD (n = 3). Asterisks (*) indicate statistically significant differences in comparison with positive control (ANOVA test), where **** = *p* < 0.0001, ** = *p* < 0.01, * = *p* < 0.05)

**Table 10:** Minimum inhibitory concentration (MIC) values of the OHEs on the tested strains.

Bacterial Strains	OHEs	Concentration mg/mL											
		100	50	25	12.5	6.25	3.12	1.6	0.8	0.4	0.2	0.1	C+
*S. aureus* MSSA ATCC 25923	*Sambucus nigra*	−	−	−	+	+	+	+	+	+	+	+	+
	*Epilobium hirsutum*	−	−	−	−	+	+	+	+	+	+	+	+
	*Lythrum salicaria*	−	−	−	−	−	+	+	+	+	+	+	+
*S. aureus* MRSA ATCC 700699	*Sambucus nigra*	−	−	+	+	+	+	+	+	+	+	+	+
	*Epilobium hirsutum*	−	−	−	−	+	+	+	+	+	+	+	+
	*Lythrum salicaria*	−	−	−	−	−	+	+	+	+	+	+	+
*E. coli* ATCC 25922	*Sambucus nigra*	−	−	−	+	+	+	+	+	+	+	+	+
	*Epilobium hirsutum*	−	−	−	−	−	+	+	+	+	+	+	+
	*Lythrum salicaria*	−	−	−	−	+	+	+	+	+	+	+	+
*P. aeruginosa* ATCC 27853	*Sambucus nigra*	−	−	−	+	+	+	+	+	+	+	+	+
	*Epilobium hirsutum*	−	−	−	+	+	+	+	+	+	+	+	+
	*Lythrum salicaria*	−	−	−	−	+	+	+	+	+	+	+	+

C+ positive control; (+) presence of growth, (−) absence of growth.

**Table 11:** Non-toxic concentrations of the OHEs.

Levels	*Sambucus nigra*	*Lythrum salicaria*	*Epilobium hirsutum*
	Non-toxic concentrations of OHEs on HaCaT (µg/mL)		
H	400	150	50
M	100	75	25
L	25	25	1
	Non-toxic concentrations of OHEs on BJ (µg/mL)		
H	400	50	25
M	100	25	10
L	25	1	1
	Non-toxic concentrations of OHEs on RAW 264.7 (µg/mL)		
H	400	50	150
M	100	25	75
L	25	1	25

H—high, M—medium, L—low concentrations.

**Table 12:** Wound closure and cell migration of HaCaT and BJ after treatment with the OHEs for 24 h.

Wound Closure After 24 h (%)			Cell Migration After 24 h (mm/h)	
OHEs	HaCaT	BJ	HaCaT	BJ
*Sambucus nigra*	59.87 ± 13.33 *	53.62 ± 11.70	4.50 ± 0.83 *	3.85 ± 1.76
*Lythrum salicaria*	49.54 ± 5.13	40.16 ± 16.34	3.97 ± 0.67	4.26 ± 1.44
*Epilobium hirsutum*	98.49 ± 2.60 ***	76.49 ± 8.20 *	5.94 ± 0.64 *	4.66 ± 1.73
NC	44.01 ± 17.32	37.31 ± 6.25	3.26 ± 1.31	4.29 ± 3.54

NC—negative control (medium). Data are expressed as mean ± SD (n ≥ 3). Asterisks (*) indicate statistically significant differences in comparison with positive control (ANOVA test), where *** = *p* < 0.001, * = *p* < 0.05.

## Data Availability

The original contributions presented in this study are included in the article/Supplementary Material. Further inquiries can be directed to the corresponding author.

## Supplementary Materials

The following supporting information can be downloaded at: https://www.mdpi.com/article/10.3390/antiox14050521/s1, Figure S1. Ishikawa diagram for the preparation of HEs; Table S1. Equations of the calibration curves for the polyphenols analyzed with the first analytical method and the limits of detection (LOD) and quantification (LOQ), respectively; Table S2. Equations of the calibration curves for the polyphenols analyzed with the second analytical method and the limits of detection (LOD) and quantification (LOQ), respectively; Table S3. Equations of the calibration curves for the analyzed sterols and the limits of detection (LOD) and quantification (LOQ), respectively; Table S4. Equations of the calibration curves for the analyzed tocopherols and the limits of detection (LOD) and quantification (LOQ), respectively; Table S5. Equations of the calibration curves for the analyzed procyanidins and the limits of detection (LOD) and quantification (LOQ), respectively; Table S6. FMEA risk assessment on the quality of the HEs.

## Abbreviations

HEs	Herbal extracts of *Sambucus nigra*, *Epilobium hirsutum*, and *Lythrum salicaria* from this study before optimization or other extracts from these plant materials previously reported in the literature when used for comparison
OHEs	Optimized herbal extracts of *Sambucus nigra*, *Epilobium hirsutum*, and *Lythrum salicaria* after being optimized in this study
UTE	Ultra-turrax-assisted extraction
USE	Ultrasound-assisted extraction
TPC	Total polyphenol content, mg GAE/mL HE = mg gallic acid equivalents/mL of HE
TFC	Total flavonoid content, mM QAE/mL HE = mM quercetin equivalents/mL of HE
MIC	Minimum inhibitory concentration
DPPH	2,2-diphenyl-1-picrylhydrazyl radical scavenging capacity, Trolox equivalents (TEs) mg/mL of OHE.
FRAP	Ferric reducing antioxidant power, Trolox equivalents (TEs) mM/OHE.
TEAC	Trolox equivalent antioxidant capacity, Trolox equivalents (TEs) mM/OHE.

## References

[1] Hoang H.T., Moon J.Y., Lee Y.C. (2021). Natural Antioxidants from Plant Extracts in Skincare Cosmetics: Recent Applications, Challenges and Perspectives. Cosmetics.

[2] Michalak M. (2023). Plant Extracts as Skin Care and Therapeutic Agents. Int. J. Mol. Sci..

[3] Safta D.A., Bogdan C., Moldovan M.L. (2022). Vesicular Nanocarriers for Phytocompounds in Wound Care: Preparation and Characterization. Pharmaceutics.

[4] Safta D.A., Bogdan C., Moldovan M.L. (2024). SLNs and NLCs for Skin Applications: Enhancing the Bioavailability of Natural Bioactives. Pharmaceutics.

[5] Pathak D., Mazumder A. (2024). A critical overview of challenging roles of medicinal plants in improvement of wound healing technology. DARU J. Pharm. Sci..

[6] Trinh X.-T., Long N.-V., Van Anh L.T., Nga P.T., Giang N.N., Chien P.N., Nam S.-Y., Heo C.-Y. (2022). A Comprehensive Review of Natural Compounds for Wound Healing: Targeting Bioactivity Perspective. Int. J. Mol. Sci..

[7] Hosseinkhani A., Falahatzadeh M., Raoofi E., Zarshenas M.M. (2016). An Evidence-Based Review on Wound Healing Herbal Remedies From Reports of Traditional Persian Medicine. J. Evid. Based Complement. Altern. Med..

[8] Schoukens G., Rajendran S. (2019). Bioactive dressings to promote wound healing. Advanced Textiles for Wound Care.

[9] Wilkinson H.N., Hardman M.J. (2020). Wound healing: Cellular mechanisms and pathological outcomes. Open Biol..

[10] Rodrigues M., Kosaric N., Bonham C.A., Gurtner G.C. (2019). Wound healing: A cellular perspective. Physiol. Rev..

[11] Balderas-Cordero D., Canales-Alvarez O., Sánchez-Sánchez R., Cabrera-Wrooman A., Canales-Martinez M.M., Rodriguez-Monroy M.A. (2023). Anti-Inflammatory and Histological Analysis of Skin Wound Healing through Topical Application of Mexican Propolis. Int. J. Mol. Sci..

[12] Allaw M., Manca M.L., Gómez-Fernández J.C., Pedraz J.L., Terencio M.C., Sales O.D., Nacher A., Manconi M. (2021). Oleuropein Multicompartment Nanovesicles Enriched with Collagen as A Natural Strategy for the Treatment of Skin Wounds Connected with Oxidative Stress. Nanomedicine.

[13] Nasab M.E., Takzaree N., Saffaria P.M., Partoazar A. (2019). In vitro antioxidant activity and in vivo wound-healing effect of lecithin liposomes: A comparative study. J. Comp. Eff. Res..

[14] Pandey V.K., Ajmal G., Upadhyay S.N., Mishra P.K. (2020). Nano-fibrous scaffold with curcumin for anti-scar wound healing. Int. J. Pharm..

[15] Manconi M., Manca M.L., Marongiu F., Caddeo C., Castangia I., Petretto G.L., Pintoreb G., Saraisa G., D’hallewinc G., Zarud M. (2016). Chemical characterization of *Citrus limon* var. pompia and incorporation in phospholipid vesicles for skin delivery. Int. J. Pharm..

[16] Moulaoui K., Caddeo C., Manca M.L., Castangia I., Valenti D., Escribano E., Atmani D., Fadda A.M., Manconi M. (2015). Identification and nanoentrapment of polyphenolic phytocomplex from Fraxinus angustifolia: In vitro and in vivo wound healing potential. Eur. J. Med. Chem..

[17] Breijyeh Z., Karaman R. (2024). Antibacterial activity of medicinal plants and their role in wound healing. Future J. Pharm. Sci..

[18] Kassam N.A., Damian D.J., Kajeguka D., Nyombi B., Kibiki G.S. (2017). Spectrum and antibiogram of bacteria isolated from patients presenting with infected wounds in a Tertiary Hospital, northern Tanzania. BMC Res. Notes.

[19] Mirhaj M., Labbaf S., Tavakoli M., Seifalian A. (2022). An Overview on the Recent Advances in the Treatment of Infected Wounds: Antibacterial Wound Dressings. Macromol. Biosci..

[20] Arip M., Selvaraja M., Mogana R., Tan L.F., Leong M.Y., Tan P.L., Yap V.L., Chinnapan S., Tat N.C., Abdullah M. (2022). Review on Plant-Based Management in Combating Antimicrobial Resistance-Mechanistic Perspective. Front. Pharmacol..

[21] Srivastava J., Chandra H., Nautiyal A.R., Kalra S.J.S. (2014). Antimicrobial resistance (AMR) and plant-derived antimicrobials (PDAms) as an alternative drug line to control infections. Biotech.

[22] Sibanda T., Okoh A.I. (2007). The challenges of overcoming antibiotic resistance: Plant extracts as potential sources of antimicrobial and resistance modifying agents. Afr. J. Biotechnol..

[23] Safta D.A., Ielciu I., Șuștic R., Hanganu D., Niculae M., Cenariu M., Pall E., Moldovan M.L., Achim M., Bogdan C. (2023). Chemical Profile and Biological Effects of an Herbal Mixture for the Development of an Oil-in-Water Cream. Plants.

[24] Vlase A.M., Toiu A., Tomuță I., Vlase L., Muntean D., Casian T., Fizesan I., Nadas G.C., Novac C.Ș., Tamas M. (2023). Epilobium Species: From Optimization of the Extraction Process to Evaluation of Biological Properties. Antioxidants.

[25] Solcan M.B., Fizeșan I., Vlase L., Vlase A.M., Rusu M.E., Mateș L., Petru A.-E., Creștin I.-V., Tomuță I., Popa D.-S. (2023). Phytochemical Profile and Biological Activities of Extracts Obtained from Young Shoots of Blackcurrant (*Ribes nigrum* L.), European Blueberry (*Vaccinium myrtillus* L.), and Mountain Cranberry (*Vaccinium vitis-idaea* L.). Horticulturae.

[26] Bogdan C., Safta D.A., Iurian S., Petrușcă D.R., Moldovan M.L. (2024). QbD Approach in Cosmetic Cleansers Research: The Development of a Moisturizing Cleansing Foam Focusing on Thickener, Surfactants, and Polyols Content. Gels.

[27] Skowrońska W., Granica S., Piwowarski J.P., Jakupović L., Zovko Končić M., Bazylko A. (2024). Wound healing potential of extract from *Sambucus nigra* L. leaves and its fractions. J. Ethnopharmacol..

[28] Sala G., Pasta S., Maggio A., La Mantia T. (2023). *Sambucus nigra* L. (fam. Viburnaceae) in Sicily: Distribution, Ecology, Traditional Use and Therapeutic Properties. Plants.

[29] Tiboc Schnell C.N., Filip G.A., Decea N., Moldovan R., Opris R., Man S.C., Moldovan B., David L., Tabăran F., Olteanu D. (2021). The impact of *Sambucus nigra* L. extract on inflammation, oxidative stress and tissue remodeling in a rat model of lipopolysaccharide-induced subacute rhinosinusitis. Inflammopharmacology.

[30] Wójciak M., Ziemlewska A., Zagórska-Dziok M., Nizioł-Łukaszewska Z., Szczepanek D., Oniszczuk T., Sowa I. (2023). Anti-Inflammatory and Protective Effects of Water Extract and Bioferment from Sambucus nigra Fruit in LPS-Induced Human Skin Fibroblasts. Int. J. Mol. Sci..

[31] Studzińska-Sroka E., Paczkowska-Walendowska M., Woźna Z., Plech T., Szulc P., Cielecka-Piontek J. (2024). Elderberry Leaves with Antioxidant and Anti-Inflammatory Properties as a Valuable Plant Material for Wound Healing. Pharmaceuticals.

[32] Seymenska D., Teneva D., Nikolova I., Benbassat N., Denev P. (2024). In Vivo Anti-Inflammatory and Antinociceptive Activities of Black Elder (*Sambucus nigra* L.) Fruit and Flower Extracts. Pharmaceuticals.

[33] Stępień A.E., Trojniak J., Tabarkiewicz J. (2023). Health-Promoting Properties: Anti-Inflammatory and Anticancer Properties of *Sambucus nigra* L. Flowers and Fruits. Molecules.

[34] Santin J.R., Benvenutti L., Broering M.F., Nunes R., Goldoni F.C., Patel Y.B.K., de Souza J.A., Kopp M.A.T., de Souza P., da Silva R.d.C.V. (2022). Sambucus nigra: A traditional medicine effective in reducing inflammation in mice. J. Ethnopharmacol..

[35] Laurutis A., Liobikas J., Stanciauskaite M., Marksa M., Ramanauskiene K., Majiene D. (2022). Comparison of the Formulation, Stability and Biological Effects of Hydrophilic Extracts from Black Elder Flowers (*Sambucus nigra* L.). Pharmaceutics.

[36] Karakaya S., Süntar I., Yakinci O.F., Sytar O., Ceribasi S., Dursunoglu B., Ozbek H., Guvenalp Z. (2020). In vivo bioactivity assessment on Epilobium species: A particular focus on Epilobium angustifolium and its components on enzymes connected with the healing process. J. Ethnopharmacol..

[37] Nowak A., Zagórska-Dziok M., Ossowicz-Rupniewska P., Makuch E., Duchnik W., Kucharski Ł., Adamiak-Giera U., Prowans P., Czapla N., Bargiel P. (2021). *Epilobium angustifolium* L. Extracts as Valuable Ingredients in Cosmetic and Dermatological Products. Molecules.

[38] Nowak A., Zagórska-Dziok M., Perużyńska M., Cybulska K., Kucharska E., Ossowicz-Rupniewska P., Piotrowska K., Duchnik W., Kucharski Ł., Sulikowski T. (2022). Assessment of the Anti-Inflammatory, Antibacterial and Anti-Aging Properties and Possible Use on the Skin of Hydrogels Containing *Epilobium angustifolium* L. Extracts. Front. Pharmacol..

[39] Nowak A., Duchnik W., Makuch E., Kucharski Ł., Ossowicz-rupniewska P., Cybulska K., Sulikowski T., Moritz M., Klimowicz A. (2021). *Epilobium angustifolium* L. Essential Oil—Biological Activity and Enhancement of the Skin Penetration of Drugs—In Vitro Study. Molecules.

[40] Kiss A.K., Bazylko A., Filipek A., Granica S., Jaszewska E., Kiarszys U., Kośmider A., Piwowarski J. (2011). Oenothein B’s contribution to the anti-inflammatory and antioxidant activity of *Epilobium* sp.. Phytomedicine.

[41] Ak G., Zengin G., Mahomoodally M.F., Llorent-Martínez E., Orlando G., Chiavaroli A., Brunetti L., Recinella L., Leone S., Di Simone S.C. (2021). Shedding light into the connection between chemical components and biological effects of extracts from *Epilobium hirsutum*: Is it a potent source of bioactive agents from natural treasure?. Antioxidants.

[42] Nowak A., Zielonka-Brzezicka J., Perużyńska M., Klimowicz A. (2022). *Epilobium angustifolium* L. as a Potential Herbal Component of Topical Products for Skin Care and Treatment—A Review. Molecules.

[43] Piwowarski J.P., Kiss A.K. (2014). Contribution of C-glucosidic ellagitannins to *Lythrum salicaria* L. influence on pro-inflammatory functions of human neutrophils. J. Nat. Med..

[44] Piwowarski J.P., Granica S., Kiss A.K. (2015). *Lythrum salicaria* L.—Underestimated medicinal plant from European traditional medicine. A review. J. Ethnopharmacol..

[45] Tunalier Z., Koşar M., Küpeli E., Çaliş I., Başer K.H.C. (2007). Antioxidant, anti-inflammatory, anti-nociceptive activities and composition of *Lythrum salicaria* L. extracts. J. Ethnopharmacol..

[46] Vafi F., Bahramsoltani R., Abdollahi M., Manayi A., Abdolghaffari A.H., Samadi N., Amin G., Hassanzadeh G., Jamalifar H., Baeeri M. (2016). Burn Wound Healing Activity of *Lythrum salicaria* L. and *Hypericum scabrum* L.. Index Wounds.

[47] Jouravel G., Guénin S., Bernard F.X., Elfakir C., Bernard P., Himbert F. (2017). New Biological Activities of *Lythrum salicaria* L.: Effects on Keratinocytes, Reconstructed Epidermis and Reconstructed Skins, Applications in Dermo-Cosmetic Sciences. Cosmetics.

[48] Mykhailenko O., Ivanauskas L., Bezruk I., Petrikaitė V., Georgiyants V. (2022). Application of Quality by Design Approach to the Pharmaceutical Development of Anticancer Crude Extracts of Crocus sativus Perianth. Sci. Pharm..

[49] Pașca D., Frangiamone M., Mangiapelo L., Vila-Donat P., Mîrza O., Vlase A.M., Miere D., Filip L., Mañes J., Loghin F. (2024). Phytochemical Characterization of Bilberries and Their Potential as a Functional Ingredient to Mitigate Ochratoxin A Toxicity in Cereal-Based Products. Nutrients.

[50] Csepregi K., Neugart S., Schreiner M., Hideg É. (2016). Comparative evaluation of total antioxidant capacities of plant polyphenols. Molecules.

[51] Pinacho R., Cavero R.Y., Astiasarán I., Ansorena D., Calvo M.I. (2015). Phenolic compounds of blackthorn (*Prunus spinosa* L.) and influence of in vitro digestion on their antioxidant capacity. J. Funct. Foods.

[52] Gligor O., Clichici S., Moldovan R., Muntean D., Vlase A.M., Nadăș G.C., Matei I.A., Filip G.A., Vlase L., Crișan G. (2023). The Effect of Extraction Methods on Phytochemicals and Biological Activities of Green Coffee Beans Extracts. Plants.

[53] Benedec D., Oniga I., Hanganu D., Gheldiu A.M., Puşcaş C., Silaghi-Dumitrescu R., Duma M., Tiperciuc B., Vârban R., Vlase L. (2018). Sources for developing new medicinal products: Biochemical investigations on alcoholic extracts obtained from aerial parts of some Romanian Amaryllidaceae species. BMC Complement. Altern. Med..

[54] Carpa R., Drăgan-Bularda M., Muntean V. (2014). Microbiologie Generala—Lucrari Practice.

[55] Ungurean C., Carpa R., Campean R., Maior M.C., Olah N.K. (2020). Phytochemical and microbial analyses of *Berberis* sp. extracts. Rom Biotechnol. Lett..

[56] Pop A., Bogdan C., Fizesan I., Iurian S., Carpa R., Bacali C., Vlase L., Benedec D., Moldovan M.L. (2022). In Vitro Evaluation of Biological Activities of Canes and Pomace Extracts from Several Varieties of *Vitis vinifera* L. for Inclusion in Freeze-Drying Mouthwashes. Antioxidants.

[57] EUCAST: MIC Determination of Non-Fastidious and Fastidious Organisms [Internet]. https://www.eucast.org/ast_of_bacteria/mic_determination.

[58] Pop A., Fizesan I., Vlase L., Rusu M.E., Cherfan J., Babota M., Gheldiu A.-M., Tomuta I., Popa D.-S. (2021). Enhanced Recovery of Phenolic and Tocopherolic Compounds from Walnut (*Juglans regia* L.) Male Flowers Based on Process Optimization of Ultrasonic Assisted-Extraction: Phytochemical Profile and Biological Activities. Antioxidants.

[59] Riss T.L., Moravec R.A., Niles A.L. (2013). Cell Viability Assays. https://www.ncbi.nlm.nih.gov/books/NBK144065/?report=reader.

[60] Rusu M.E., Fizeșan I., Pop A., Gheldiu A.M., Mocan A., Crișan G., Vlase L., Loghin F., Popa D.-S., Tomuta I. (2019). Enhanced recovery of antioxidant compounds from hazelnut (*Corylus avellana* L.) involucre based on extraction optimization: Phytochemical profile and biological activities. Antioxidants.

[61] Hwang J.H., Ma J.N., Park J.H., Jung H.W., Park Y.-K. (2018). Anti-inflammatory and antioxidant effects of MOK, a polyherbal extract, on lipopolysaccharide-stimulated RAW 264.7 macrophages. Int. J. Mol. Med..

[62] Suarez-Arnedo A., Figueroa F.T., Clavijo C., Arbeláez P., Cruz J.C., Muñoz-Camargo C. (2020). An image J plugin for the high throughput image analysis of in vitro scratch wound healing assays. PLoS ONE.

[63] Cedillo-Cortezano M., Martinez-Cuevas L.R., López J.A.M., Barrera López I.L., Escutia-Perez S., Petricevich V.L. (2024). Use of Medicinal Plants in the Process of Wound Healing: A Literature Review. Pharmaceuticals.

[64] Qazimi B., Stanoeva J.P., Cvetanoska M., Geskovski N., Dragusha S., Koraqi H., Qazimi V., Ejupi V. (2023). Phenolic Compound Composition of *Sambucus nigra* L. Wild-Growing Plants from Kosovo. Turk. J. Pharm. Sci..

[65] Muhamad I.I., Hassan N.D., Mamat S.N.H., Nawi N.M., Rashid W.A., Tan N.A. (2016). Extraction Technologies and Solvents of Phytocompounds from Plant Materials: Physicochemical Characterization and Identification of Ingredients and Bioactive Compounds from Plant Extract Using Various Instrumentations. Ingredients Extraction by Physicochemical Methods in Food Handbook of Food Bioengineering.

[66] Xu W.J., Zhai J.W., Cui Q., Liu J.Z., Luo M., Fu Y.J., Zu Y.-G. (2016). Ultra-turrax based ultrasound-assisted extraction of five organic acids from honeysuckle (*Lonicera japonica* Thunb.) and optimization of extraction process. Sep. Purif. Technol..

[67] Rusu M.E., Gheldiu A.M., Mocan A., Moldovan C., Popa D.S., Tomuta I., Vlase L. (2018). Process optimization for improved phenolic compounds recovery from walnut (*Juglans regia* L.) Septum: Phytochemical profile and biological activities. Molecules.

[68] Domínguez R., Zhang L., Rocchetti G., Lucini L., Pateiro M., Munekata P.E.S., Lorenzo J.M. (2020). Elderberry (*Sambucus nigra* L.) as potential source of antioxidants. Characterization, optimization of extraction parameters and bioactive properties. Food Chem..

[69] Pereira D.I., Amparo T.R., Almeida T.C., Costa F.S.F., Brandão G.C., dos Santos O.D.H., da Silva G.N., Bianco de Souza G.H. (2022). Cytotoxic activity of butanolic extract from *Sambucus nigra* L. flowers in natura and vehiculated in micelles in bladder cancer cells and fibroblasts. Nat. Prod. Res..

[70] Chen L.Y., Huang C.N., Liao C.K., Chang H.M., Kuan Y.H., Tseng T.J., Yen K.-J., Yang K.-L., Lin H.-C. (2020). Effects of rutin on wound healing in hyperglycemic rats. Antioxidants.

[71] Moghadam S.E., Ebrahimi S.N., Salehi P., Farimani M.M., Hamburger M., Jabbarzadeh E. (2017). Wound healing potential of chlorogenic acid and myricetin-3-o-β-rhamnoside isolated from parrotia persica. Molecules.

[72] Song L., Yang H., Liang D., Chu D., Yang L., Li M., Yang B., Shi Y., Chen Z., Yu Z. (2022). A chlorogenic acid-loaded hyaluronic acid-based hydrogel facilitates anti-inflammatory and pro-healing effects for diabetic wounds. J. Drug Deliv. Sci. Technol..

[73] Bagdas D., Gul N.Y., Topal A., Tas S., Ozyigit M.O., Cinkilic N., Gul Z., Cam Etoz B., Ziyanok S., Inan S. (2014). Pharmacologic overview of systemic chlorogenic acid therapy on experimental wound healing. Naunyn Schmiedebergs Arch. Pharmacol..

[74] Chen W.C., Liou S.S., Tzeng T.F., Lee S.L., Liu I.M. (2013). Effect of topical application of chlorogenic acid on excision wound healing in rats. Planta Med..

[75] Gómez-Florit M., Monjo M., Ramis J.M. (2014). Identification of Quercitrin as a Potential Therapeutic Agent for Periodontal Applications. J. Periodontol..

[76] Gómez-Florit M., Monjo M., Ramis J.M. (2015). Quercitrin for periodontal regeneration: Effects on human gingival fibroblasts and mesenchymal stem cells. Sci. Rep..

[77] Bhatia N., Kaur G., Soni V., Kataria J., Dhawan R.K. (2016). Evaluation of the wound healing potential of isoquercetin-based cream on scald burn injury in rats. Burn. Trauma.

[78] Hoff J., Karl B., Gerstmeier J., Beekmann U., Schmölz L., Börner F., Kralisch D., Bauer M., Werz O., Fischer D. (2021). Controlled release of the α-tocopherol-derived metabolite α-13′-carboxychromanol from bacterial nanocellulose wound cover improves wound healing. Nanomaterials.

[79] Ghahremani-Nasab M., Akbari-Gharalari N., Rahmani Del Bakhshayesh A., Ghotaslou A., Ebrahimi-kalan A., Mahdipour M., Mehdipour A. (2023). Synergistic effect of chitosan-alginate composite hydrogel enriched with ascorbic acid and alpha-tocopherol under hypoxic conditions on the behavior of mesenchymal stem cells for wound healing. Stem Cell Res. Ther..

[80] Ehterami A., Salehi M., Farzamfar S., Samadian H., Vaez A., Ghorbani S., Ai J., Sahrapeyma H. (2019). Chitosan/alginate hydrogels containing Alpha-tocopherol for wound healing in rat model. J. Drug Deliv. Sci. Technol..

[81] Na Y., Woo J., Choi WIl Lee J.H., Hong J., Sung D. (2021). α-Tocopherol-loaded reactive oxygen species-scavenging ferrocene nanocapsules with high antioxidant efficacy for wound healing. Int. J. Pharm..

[82] Liang Q., Yang J., He J., Chen X., Zhang H., Jia M., Liu K., Jia C., Pan Y., Wei J. (2020). Stigmasterol alleviates cerebral ischemia/reperfusion injury by attenuating inflammation and improving antioxidant defenses in rats. Biosci. Rep..

[83] Bakrim S., Benkhaira N., Bourais I., Benali T., Lee L.H., El Omari N., Sheikh R.A., Goh K.W., Ming L.C., Bouyahya A. (2022). Health Benefits and Pharmacological Properties of Stigmasterol. Antioxidants.

[84] Nazir S., Ahmad I., Mobashar A., Sharif A., Shabbir A., Chaudhary W.A. (2023). Mechanistic evaluation of antiarthritic and anti-inflammatory effect of campesterol ester derivatives in complete Freund’s adjuvant-induced arthritic rats. Front. Pharmacol..

[85] Khan Z., Nath N., Rauf A., Emran TBin Mitra S., Islam F., Chandran D., Barua J., Khandaker M.U., Idris A.M., Wilairatana P. (2022). Multifunctional roles and pharmacological potential of β-sitosterol: Emerging evidence toward clinical applications. Chem. Biol. Interact..

[86] Baskar A.A., Al Numair K.S., Gabriel Paulraj M., Alsaif M.A., Muamar M.A., Ignacimuthu S. (2012). β-sitosterol prevents lipid peroxidation and improves antioxidant status and histoarchitecture in rats with 1,2-dimethylhydrazine-induced colon cancer. J. Med. Food.

[87] Vivancos M., Moreno J.J. (2005). β-Sitosterol modulates antioxidant enzyme response in RAW 264.7 macrophages. Free Radic. Biol. Med..

[88] Gupta R., Sharma A.K., Dobhal M.P., Sharma M.C., Gupta R.S. (2011). Antidiabetic and antioxidant potential of β-sitosterol in streptozotocin-induced experimental hyperglycemia. J. Diabetes.

[89] Hammam W.E., Gad A.M., Gad M.K., Kirollos F.N., Yassin N.A., Tantawi M.E.E., El Hawary S.S.E. (2022). *Pyrus communis* L. (Pear) and *Malus domestica* Borkh. (apple) leaves lipoidal extracts as sources for beta-sitosterol rich formulae and their wound healing evaluation. Nat. Prod. Res..

[90] Dighe S.B., Kuchekar B.S., Wankhede S.B., Santosh M., Dighe Head B. (2016). Analgesic and anti-inflammatory activity of β-sitosterol isolated from leaves of *Oxalis corniculata*. Int. J. Pharmacol. Res..

[91] Ododo M.M., Choudhury M.K., Dekebo A.H. (2016). Structure elucidation of β-sitosterol with antibacterial activity from the root bark of Malva parviflora. Springerplus.

[92] Petkova D.T., Mihaylova D.S., Deseva I.N., Denev P.N., Krastanov A.I. (2021). Green approach to obtain extracts of seven edible flowers. IOP Conf. Ser. Mater. Sci. Eng..

[93] Tundis R., Ursino C., Bonesi M., Loizzo M.R., Sicari V., Pellicanò T., Manfredi I.L., Figoli A., Cassano A. (2019). Flower and leaf extracts of *Sambucus nigra* L.: Application of membrane processes to obtain fractions with antioxidant and antityrosinase properties. Membranes.

[94] Dawidowicz A.L., Wianowska D., Baraniak B. (2006). The antioxidant properties of alcoholic extracts from *Sambucus nigra* L. (antioxidant properties of extracts). LWT.

[95] Jamshidi M., Shabani E., Hashemi Z., Ebrahimzadeh M.A. (2014). Evaluation of three methods for the extraction of antioxidants from leaf and aerial parts of *Lythrum salicaria* L. (Lythraceae). Int. Food Res. J..

[96] Kustova T., Karpenyuk T., Goncharova A., Mamonov L., Ross S. (2014). Herbal extracts in the treatment of Diabetic Foot Syndrome. Cent. Asian J. Glob. Health.

[97] Castangia I., Nácher A., Caddeo C., Valenti D., Fadda A.M., Díez-Sales O., Ruiz-Saurí A., Manconi M. (2014). Fabrication of quercetin and curcumin bionanovesicles for the prevention and rapid regeneration of full-thickness skin defects on mice. Acta Biomater..

[98] Milkova-Tomova I., Kazakova Z., Buhalova D., Gentscheva G., Nikolova K., Minkova S. (2023). Antioxidant Properties and Antibacterial Activity of Water Extracts from *Sambucus nigra* L. under Different Conditions. Folia Med..

[99] Ferreira-Santos P., Badim H., Salvador Â.C., Silvestre A.J.D., Santos S.A.O., Rocha S.M., Sousa A.M., Pereira M.O., Wilson C.P., Rocha C.M.R. (2021). Chemical characterization of *Sambucus nigra* L. Flowers aqueous extract and its biological implications. Biomolecules.

[100] Haș I.M., Teleky B.E., Szabo K., Simon E., Ranga F., Diaconeasa Z.M., Purza A.L., Vodnar D.-C., Tit D.M., Nițescu M. (2023). Bioactive Potential of Elderberry (*Sambucus nigra* L.): Antioxidant, Antimicrobial Activity, Bioaccessibility and Prebiotic Potential. Molecules.

[101] Przybylska-Balcerek A., Szablewski T., Szwajkowska-Michałek L., Świerk D., Cegielska-Radziejewska R., Krejpcio Z., Suchowilska E., Tomczyk Ł., Stuper-Szablewska K. (2021). *Sambucus nigra* extracts–natural antioxidants and antimicrobial compounds. Molecules.

[102] Hearst C., Mccollum G., Nelson D., Ballard L.M., Millar B.C., Goldsmith C.E., Rooney P.J., Loughrey A., Moore J.E., Rao J.R. (2010). Antibacterial activity of elder (*Sambucus nigra* L.) flower or berry against hospital pathogens. J. Med. Plants Res..

[103] Rauha J.-P., Remes S., Heinonen M., Hopia A., Kähkönen M., Kujala T., Pihlaja K., Vuorela H., Vuorela P. (2000). Antimicrobial effects of Finnish plant extracts containing flavonoids and other phenolic compounds. Int. J. Food Microbiol..

[104] Becker H., Scher J.M., Speakman J.B., Zapp J. (2005). Bioactivity guided isolation of antimicrobial compounds from *Lythrum salicaria*. Fitoterapia.

[105] Wyse D., Fulcher R.G., Ehlke N.J., Biesboer D. (2008). Antimicrobial activity of native and naturalized plants of Minnesota and Wisconsin. J. Med. Plants Res..

[106] Guclu E., Genc H., Zengin M., Karabay O. (2014). Antibacterial Activity of *Lythrum salicaria* against Multidrug-resistant *Acinetobacter baumannii* and *Pseudomonas aeruginosa*. Annu. Res. Rev. Biol..

[107] Battinelli L., Tita B., Evandri M.G., Mazzanti G. (2001). Antimicrobial activity of Epilobium spp. extracts. Farmaco.

[108] Gonzalez-Pastor R., Carrera-Pacheco S.E., Zúñiga-Miranda J., Rodríguez-Pólit C., Mayorga-Ramos A., Guamán L.P., Barba-Ostria C. (2023). Current Landscape of Methods to Evaluate Antimicrobial Activity of Natural Extracts. Molecules.

[109] Lin P., Hwang E., Ngo H.T.T., Seo S.A., Yi T.H. (2019). *Sambucus nigra* L. ameliorates UVB-induced photoaging and inflammatory response in human skin keratinocytes. Cytotechnology.

[110] Filip G.A., Florea A., Olteanu D., Clichici S., David L., Moldovan B., Cenariu M., Scrobota I., Potara M., Baldea I. (2021). Biosynthesis of silver nanoparticles using *Sambucus nigra* L. fruit extract for targeting cell death in oral dysplastic cells. Mater. Sci. Eng. C.

[111] Skowrońska W., Granica S., Czerwińska M.E., Osińska E., Bazylko A. (2022). *Sambucus nigra* L. leaves inhibit TNF-α secretion by LPS-stimulated human neutrophils and strongly scavenge reactive oxygen species. J. Ethnopharmacol..

[112] Zielińska-Wasielica J., Olejnik A., Kowalska K., Olkowicz M., Dembczyński R. (2019). Elderberry (*Sambucus nigra* L.) fruit extract alleviates oxidative stress, insulin resistance, and inflammation in hypertrophied 3T3-L1 adipocytes and activated RAW 264.7 macrophages. Foods.

[113] Ferreira S.S., Martins-Gomes C., Nunes F.M., Silva A.M. (2022). Elderberry (*Sambucus nigra* L.) extracts promote anti-inflammatory and cellular antioxidant activity. Food Chem. X.

[114] Kim H.Y., Lim S.H., Park M.H., Park Y.H., Ham H.J., Lee K.Y., Park D.S., Kim K.H., Kim S.M. (2010). Biological Activities in the Leaf Extract of *Lythrum salicaria* L. Korean J. Med. Crop Sci..

[115] Merighi S., Travagli A., Tedeschi P., Marchetti N., Gessi S. (2021). Antioxidant and antiinflammatory effects of *Epilobium parviflorum*, *Melilotus officinalis* and *Cardiospermum halicacabum* plant extracts in macrophage and microglial cells. Cells.

