# Supplementary material for: Optimized Sambucus nigra L., Epilobium hirsutum L., and Lythrum salicaria L. Extracts: Biological Effects Supporting Their Potential in Wound Care
Source: Antioxidants (Basel). 2025 Apr 27;14(5):521. doi: 10.3390/antiox14050521 (PMC12108421; doi:10.3390/antiox14050521)
Supplement: Supplementary file 1 [file antioxidants-14-00521-s001.zip › antioxidants-3551226-supplementary.pdf]

antioxidants

MDPI

Supplementary file

Optimized *Sambucus nigra* L., *Epilobium hirsutum* L., and *Lythrum salicaria* L. Extracts: Biological Effects Supporting Their Potential in Wound Care

Diana Antonia Safta [1], Ana-Maria Vlase [2,*], Anca Pop [3], Julien Cherfan [4], Rahela Carpa [5], Sonia Iurian [6], Cătălina Bogdan [1,7], Laurian Vlase [6] and Mirela L. Moldovan [1]

1 Department of Dermopharmacy and Cosmetics, Faculty of Pharmacy, "Iuliu Haţieganu" University of Medicine and Pharmacy, 12 I. Creangă St. 400010 Cluj-Napoca, Romania; diana.an.safta@elearn.umfcluj.ro (D.A.S.); catalina.bogdan@umfcluj.ro (C.B.); mmoldovan@umfcluj.ro (M.L.M.)
2 Department of Pharmaceutical Botany, Faculty of Pharmacy, "Iuliu Haţieganu" University of Medicine and Pharmacy, 8 Victor Babes Street, 400012 Cluj-Napoca, Romania
3 Department of Toxicology, Faculty of Pharmacy, "Iuliu Haţieganu" University of Medicine and Pharmacy,6 L. Pasteur Street, 400349 Cluj-Napoca, Romania; anca.pop@umfcluj.ro
4 BCBS Team (Biotechnologies et Chimie des Bioressources Pour la Santé), LIENSs Laboratory (Littoral Environment et Sociétés), UMR CNRS 7266, University of la Rochelle, 17000 La Rochelle, France; julien.cherfan@univ-lr.fr
5 Department of Molecular Biology and Biotechnology, Faculty of Biology and Geology, Babeş-Bolyai University, 1 M. Kogalniceanu Street, 400084 Cluj-Napoca, Romania; rahela.carpa@ubbcluj.ro
6 Department of Pharmaceutical Technology and Biopharmacy, Faculty of Pharmacy, "Iuliu Haţieganu" University of Medicine and Pharmacy, 41 Victor Babes Street, 400012 Cluj-Napoca, Romania; sonia.iurian@umfcluj.ro (S.I.); laurian.vlase@umfcluj.ro (L.V.)
7 Department 2, Faculty of Nursing and Health Sciences, "Iuliu Haţieganu" University of Medicine and Pharmacy, 4 L. Pasteur Street t, 400349 Cluj-Napoca, Romania
* Correspondence: gheldiu.ana@umfcluj.ro

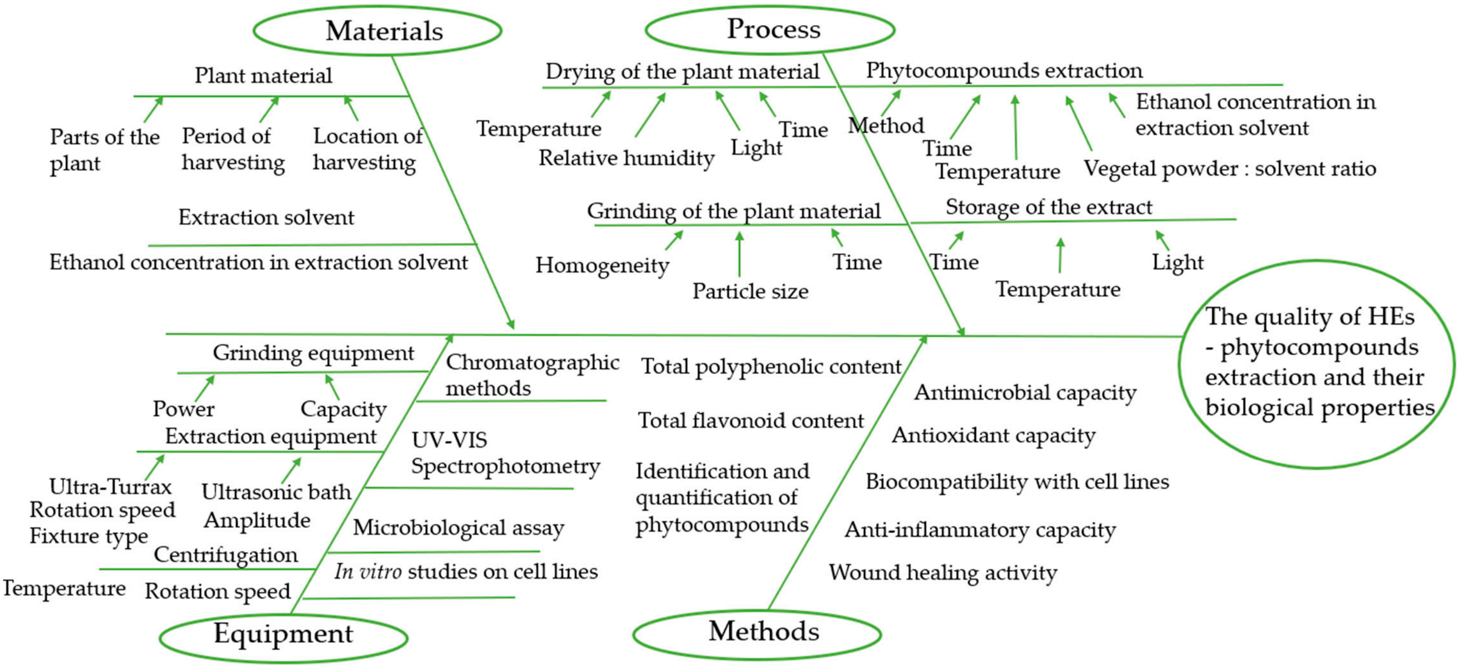

Figure S1. Ishikawa diagram for preparation of OHEs

Table S1. Equations of the calibration curves for the polyphenols analyzed with the first analytical method, the limits of detection (LOD) and quantification (LOQ), respectively

No.	Compound name	Equation of the calibration curve	LOQ (μg/mL)	LOD (μg/mL)
1	Caftaric acid	A=-0.988+25.282*x	0.2-100	0.04
2	Gentisic acid	A=-0.335+14.261*x	0.2	0.04
3	Caffeic acid	A=-0.981+45.845*x	0.2	0.04
4	Chlorogenic acid	A=-1.324+26.492*x	0.2	0.04
5	4-O-caffeoylquinic	A=0.748+26.133*x	0.2	0.04
6	p-coumaric acid	A=-0.326+33.230*x	0.2	0.04
7	Ferulic acid	A=-1.017+39.558*x	0.2	0.04
8	Sinapic acid	A=-0.237+37.103*x	0.2	0.04
9	Vitexin	A=-0.015+14.732*x	0.2	0.04
10	Hyperoside	A=0.107+19.294*x	0.2	0.04
11	Vitexin-2-O-Rhamnoside	A=0.011+10.135*x	0.2	0.04
12	Isoquercitrin	A=-0.273+12.978*x	0.2	0.04
13	Rutin	A=0.227+13.473*x	0.2	0.04
14	Myricetin	A=0.270+26.150*x	0.2	0.04
15	Fisetin	A=0.292+17.190*x	0.2	0.04
16	Quercitrin	A=0.048+10.698*x	0.2	0.04
17	Kaempferitrin	A=-0.412+12.057*x	0.2	0.04
18	Quercetol	A=-1.152+36.327*x	0.2	0.04
19	Kaempferol-3-Rhamnoside	A=0.109+12.752*x	0.2	0.04
20	Patuletin	A=-0.430+31.450*x	0.2	0.04
21	Luteolin	A=-0.761+28.927*x	0.2	0.04
22	Kaempferol	A=-1.271+30.152*x	0.2	0.04
23	Apigenin	A=-0.909+20.403*x	0.2	0.04

*A = peak area expressed in mAU´s; x = compound concentration in mg/mL

Table S2. Equations of the calibration curves for the polyphenols analysed with the second analytical method, the limits of detection (LOD) and quantification (LOQ), respectively

No.	Compound name	Equation of the calibration curve	LOQ (μg/mL)	LOD (μg/mL)
1	Epicatechin	A=1026.12+2107187*x	0.2	0.04
2	Catechin	A=94849.62+2023311*x	0.2	0.04
3	Syringic acid	A=12718.15+1242775*x	0.2	0.04
4	Gallic acid	A=6592.836+488323.7*x	0.2	0.04
5	Protocatechuic acid	A=18113.28+446436.5*x	0.2	0.04
6	Vanillic acid	A=3103.371+494246.6*x	0.2	0.04
7	Epigallocatechin	A=1178.31+745125*x	0.2	0.04
8	Epigallocatechin gallate	A=-37000+512333*x	0.2	0.04

*A = peak area expressed as MS ion abundance versus time; x = compound concentration in μg/mL

Table S3. Equations of the calibration curves for the analysed sterols, the limits of detection (LOD) and quantification (LOQ), respectively

No.	Compound name	Equation of the calibration curve	LOQ (ng/mL)	LOD (ng/mL)
1	Ergosterol	A=-554+588.8*x	80-1000	20
2	Stigmasterol	A=-15498+558.55*x	80	20
3	Beta-sitosterol	A=11298+306.3*x	80	20
4	Campesterol	A=15932+2220*x	80	20
5	Brassicasterol	A=-17955+753.55*x	80	20

*A = peak area expressed as MS ion abundance versus time; x = compound concentration in ng/mL

Table S4. Equations of the calibration curves for the analysed tocopherols, the limits of detection (LOD) and quantification (LOQ), respectively

No.	Compound name	Equation of the calibration curve	LOQ (ng/mL)	LOD (ng/mL)
1	α-tocopherol	A=-88866+3148*x	40	10
2	γ-tocopherol	A=32115+4736*x	40	10
3	δ-tocopherol	A=-3499+4071*x	40	10

*A = peak area expressed as MS ion abundance versus time; x = compound concentration in ng/mL

Table S5. Equations of the calibration curves for the analysed procyanidins, the limits of detection (LOD) and quantification (LOQ), respectively

No.	Compound name	Equation of the calibration curve	LOQ (μg/mL)	LOD (μg/mL)
1	Procyanidin A1	A=2065.204+103433.082*x	0.1	0.02
2	Procyanidin B1	A=1717.378+812606.05*x	0.1	0.02
3	Procyanidin B2	A=4179.765+1032780.8*x	0.1	0.02
4	Procyanidin B3	A=2757.94+911429.639*x	0.1	0.02
5	Procyanidin B4	A=3148.572+909225.13*x	0.1	0.02
6	Procyanidin C1	A=2262.061+174125.14*x	0.1	0.02

*A = peak area expressed as MS ion abundance versus time; x = compound concentration in μg/mL

For each compound listed in Tables 1S-5S, the accuracy and precision were within the maximum limits of ± 15% over the entire calibration range.

Table S6. FMEA risk assessment on the quality of the OHEs

Variation factors		Potential failure mode	Potential failure effects	Control method/correction action	O	S	D	RPN
**CMAs**								
**Plant material**	Part of the plant	Different concentrations in phytocompounds	Efficiency of the HEs	Separation of the relevant plant parts, botanical identification	1	4	2	8
	Period of harvesting	Different concentrations in phytocompounds	Variability of the results, efficiency of the HEs	Harvesting during the flowering time, when the concentration in phytocompounds is maximal, in the same year	1	4	1	4
	Location of harvesting	Different concentrations of phytocompounds	Variability of the results, efficiency of the HEs	Harvesting from the same location	1	2	1	2
**Extraction solvent**	Ethanol concentration in the extraction solvent	Improper extraction of phytocompounds	Efficiency of the HEs	Establishment of a proper concentration by making preliminary studies	5	5	2	**50**
**CPPs**								
**Drying of the plant material**	Temperature	Degradation of the plant material	Variability of the results, efficiency of the HEs	Drying at controlled room temperature (20±2ºC)	3	2	1	6
	Relative humidity	Degradation of the plant material	Variability of the results, efficiency of the HEs	Drying at controlled room relative humidity, safe from water sources	3	3	1	9
	Light	Degradation of the plant material	Variability of the results, efficiency of the HEs	Keeping safe from sunlight	3	3	1	9
	Time	Insufficient drying of the plant material	Efficiency of the HEs	Drying for sufficient time	3	3	2	18
**Grinding of the plant material**	Time	Improper grinding, improper extraction	Efficiency of the HEs	Grinding to obtain a homogenous powder, to ensure an efficient extraction	5	4	1	20
	Homogeneity of plant material	Improper extraction, different concentrations of phytocompounds	Variability of the results, efficiency of the HEs	Grinding to obtain a homogenous powder, to ensure an efficient extraction	4	3	1	12
	Particle size of grounded plant material	Improper extraction of phytocompounds	Efficiency of the HEs	Grinding to obtain a homogenous powder, to ensure an efficient extraction	5	4	1	16

Variation factors		Potential failure mode	Potential failure effects	Control method/correction action	O	S	D	RPN
**CMAs**								
**Phytocompounds extraction**	Method of extraction	Improper extraction of phytocompounds	Efficiency of the HEs	Establishment of a proper method by making preliminary studies	5	5	2	**50**
	Time	Improper extraction of phytocompounds	Efficiency of the HEs	Establishment of a proper time by making preliminary studies	5	5	2	**50**
	Temperature	Improper extraction/degradation of the phytocompounds	Efficiency of the HEs	Enssuring a maximum of 50 ºC to not degrade the phytocompounds	4	4	1	16
	Vegetal powder: solvent ratio	Improper extraction of phytocompounds	Efficiency of the HEs	Establishment of a proper ratio by making preliminary studies	4	4	1	16
	Ethanol concentration in extraction solvent	Improper extraction of phytocompounds	Efficiency of the HEs	Establishment of a proper concentration by making preliminary studies	5	5	2	**50**
**Storage of the extract**	Temperature	Degradation of the phytocompounds	Efficiency of the HEs	Storage in a refrigerator at 5±2 °C	4	5	1	20
	Light	Degradation of the phytocompounds	Efficiency of the HEs	Storage in a refrigerator safe from sunlight, in opaque recipients	4	4	1	16
	Time	Degradation of the phytocompounds	Stability, organoleptic properties	Storage according to the undertaken stability studies	4	3	2	24
**Equipment/characterization**								
**Grinding equipment**	Power, effectiveness, capacity	Improper grinding of the plant material	Variability of the results, efficiency of the HEs	Calibration and monitorization of the equipment, sifting through the appropriate sieve	5	5	1	25
**Extraction equipment**								
**Ultra-turrax homogenizer**	Rotation speed	Improper extraction of phytocompounds	Efficiency of the HEs	Establishment of a proper rotation speed by making preliminary studies	5	5	1	25
	Fixture type	Improper extraction of phytocompounds	Efficiency of the HEs	Establishment of a proper fixture by making preliminary studies	5	5	1	25
**Ultrasonic bath**	Amplitude of the ultrasounds	Improper extraction of phytocompounds	Efficiency of the HEs	Maintaining the same amplitude of the ultrasounds in all samples	5	5	1	25
**Centrifugation**	Rotation speed	Improper separation of the extract	Efficiency of the HEs	Establishment of a proper rotation speed by making preliminary studies	5	5	1	25
	Temperature	Degradation of the phytocompounds	Efficiency of the HEs	Setting the refrigerate centrifuge at 20 ±0.5 °c	5	5	1	25

Variation factors		Potential failure mode	Potential failure effects	Control method/correction action	O	S	D	RPN
**CMAs**								
**Characterization methods**								
**Chromatographic methods**	LOD, LOQ, sensibility, specificity	Non-reliable results	Variability of the results, efficiency of the HEs	Calibration and monitorization of the equipment and the method	5	5	2	50
**UV-VIS spectrophotometry**	LOD, LOQ, sensibility, specificity	Non-reliable results	Variability of the results, efficiency of the HEs	Calibration and monitorization of the equipment and the method	1	4	4	16
**Microbiological assay**	Culture media, incubation conditions, inoculum size, ph, temperature, microbial strain variations, risk of contamination	Non-reliable results	Variability of the results, efficiency of the HEs	Calibration and monitorization of the method, assurance of sterility	1	4	4	16
***In vitro* studies on cell lines**	Culture media, incubation conditions, ph, osmolarity, cells growth, cell passage number, cell line variations, risk of contamination	Non-reliable results	Variability of the results, efficiency of the HEs	Calibration and monitorization of the method, assurance of sterility	1	4	4	16

CMAs - critical material attributes, CPPs - critical process parameters, O - frequency of occurrence, S - severity of consequences, D - difficulty of detection, RPN - risk priority number, LOD – limit of detection, LOQ – limit of quantification.
